# Effect of Intramyocardial Grafting Collagen Scaffold With Mesenchymal Stromal Cells in Patients With Chronic Ischemic Heart Disease: A Randomized Clinical Trial

**DOI:** 10.1001/jamanetworkopen.2020.16236

**Published:** 2020-09-10

**Authors:** Xiaojun He, Qiang Wang, Yannan Zhao, He Zhang, Bin Wang, Jun Pan, Jie Li, Hongming Yu, Liudi Wang, Jianwu Dai, Dongjin Wang

**Affiliations:** 1 Department of Thoracic and Cardiovascular Surgery, the Affiliated Drum Tower Hospital of Nanjing University Medical School, Nanjing, China; 2 Key Laboratory of Molecular Developmental Biology, Institute of Genetics and Developmental Biology, Chinese Academy of Sciences, Beijing, China; 3 Center for Clinical Stem Cell Research, the Affiliated Drum Tower Hospital of Nanjing University Medical School, Nanjing, China; 4 Department of Cardiology, the Affiliated Drum Tower Hospital of Nanjing University Medical School, Nanjing, China; 5 Department of Radiology, the Affiliated Drum Tower Hospital of Nanjing University Medical School, Nanjing, China

## Abstract

**Importance:**

Cell therapy may be helpful for cardiac disease but has been fraught with poor cell retention and survival after transplantation.

**Objective:**

To determine whether cell-laden hydrogel treatment is safe and feasible for patients with chronic ischemic heart disease (CIHD).

**Design, Setting, and Participants:**

This randomized, double-blind clinical trial was conducted between March 1, 2016, and August 31, 2019, at a single hospital in Nanjing, China. Among 115 eligible patients with CIHD, 50 patients with left ventricular ejection fraction of 45% or less were selected to receive elective coronary artery bypass grafting (CABG) and additionally randomized to cell-plus-collagen treatment (collagen/cell group), cell treatment alone (cell group), or a control group. Sixty-five patients were excluded because of severe comorbidities or unwillingness to participate. Forty-four participants (88%) completed the study. The last patient completed 12 months of follow-up in August 2019. Analyses were prespecified and included all patients with available data.

**Interventions:**

During CABG, patients in the collagen/cell group were treated with human umbilical cord–derived mesenchymal stromal cell (hUC-MSC)–laden collagen hydrogel intramyocardial injection, and the cell group was treated with hUC-MSCs alone. Patients in the control group underwent CABG alone.

**Main Outcomes and Measures:**

The primary outcome was safety of the cell-laden collagen hydrogel assessed by the incidence of serious adverse events. The secondary end point was the efficacy of treatment, according to cardiovascular magnetic resonance imaging–based left ventricular ejection fraction and infarct size.

**Results:**

Fifty patients (mean [SD] age, 62.6 [8.3] years; 38 men [76%]) were enrolled, of whom 18 were randomized to the collagen/cell group, 17 to the cell group, and 15 to the control group. Patient characteristics did not differ among groups at baseline. For the primary end point, no significant differences in serious adverse events, myocardial damage markers, and renal or liver function were observed among all groups after treatment; the collagen/cell and cell groups each had 1 case of hospitalization because of heart failure, and no serious adverse events were seen in the control group. At 12 months after treatment, the mean infarct size percentage change was −3.1% (95% CI, −6.20% to −0.02%; *P* = .05) in the collagen/cell group, 5.19% (−1.85% to 12.22%, *P* = .35) in the cell group, and 8.59% (−3.06% to 20.25%, *P* = .21) in the control group.

**Conclusions and Relevance:**

This study provides, to our knowledge, the first clinical evidence that the use of collagen hydrogel is safe and feasible for cell delivery. These findings provide a basis for larger clinical studies.

**Trial Registration:**

ClinicalTrials.gov Identifier: NCT02635464

## Key Points

**Question:**

Is collagen gel a safe and feasible vehicle for cardiac cell therapy?

**Findings:**

In this randomized clinical trial that included 50 adults with chronic ischemic heart disease, patients treated with mesenchymal stromal cells in a collagen gel vehicle showed no significant difference in adverse events compared with control patients and patients treated with mesenchymal stromal cells alone. Differences between groups in scar size were not statistically significant.

**Meaning:**

Collagen gel may be a feasible and safe method to promote cell therapy; these findings thereby set the grounds for adequately powered efficacy studies.

## Teaser

This randomized clinical trial evaluates the safety and feasibility of a collagen gel vehicle for mesenchymal stromal cell treatment, compared with cell treatment alone and no cell treatment, in patients with chronic ischemic heart disease undergoing coronary artery bypass grafting.

## Introduction

There has been extensive interest in using cell-based therapy to treat patients with myocardial infarction and ischemic heart failure^1,2^ via hypothetical mechanisms involving the generation of new myocardial tissue^3,4^ or to release molecules^5^ and exosomes^6^ that harness endogenous repair mechanisms.^7^ Cells can be delivered by intramyocardial, intracoronary, and retrograde coronary venous injection to cardiac tissue. However, the cell delivery techniques used thus far have all been fraught with poor efficiency^8^ and have led to poor engraftment, retention, and survival of transplanted cells in heart tissue.^9^

Hydrogels are biomaterials designed to support delivered cells, maintain their placement in the injury zone, and enable functional integration with the injured myocardium.^10^ Collagen is the predominant protein in mammalian extracellular matrix; it provides structural support for maintaining tissue integrity and contributes to the specificity of extracellular matrix microenvironments.^11^ We developed an injectable porous collagen scaffold hydrogel derived from bovine collagen tissue. Accordingly, a randomized, single-center clinical trial was conducted to evaluate the safety and feasibility of the intramyocardial delivery of collagen hydrogel with human umbilical cord–derived mesenchymal stromal cells (hUC-MSCs) in patients with chronic ischemic heart disease (CIHD) immediately after undergoing coronary artery bypass grafting (CABG).

## Methods

### Study Design

This study was a phase 1, randomized, controlled, single-center clinical trial. The study protocol (Supplement 1) was approved by the institutional review board of the Ethics Committee of Nanjing University Medical School Affiliated Nanjing Drum Tower Hospital in China. All patients agreed to participate and signed a statement of informed consent approved by the institutional review board before enrollment. The study was performed in accordance with the principles of the Declaration of Helsinki^12^ and the Consolidated Standards of Reporting Trials (CONSORT) reporting guideline. It was conducted at the Department of Thoracic and Cardiovascular Surgery at the Affiliated Drum Tower Hospital of Nanjing University Medical School between March 1, 2016, and August 31, 2019.

### Patients

The target population was patients with CIHD with left ventricular ejection fraction (LVEF) of 45% or less (assessed by 3-D echocardiography) who needed CABG and who were not suitable for percutaneous coronary intervention revascularization.

Patients were randomized to receive hUC-MSCs (1 × 10^8^/1.5 mL phosphate-buffered saline) plus collagen scaffold (1 ml) and CABG (collagen/cell group), hUC-MSCs (1 × 10^8^/2.5 mL phosphate-buffered saline) and CABG (cell group), or CABG alone (control group). Cells and/or hydrogel were injected intramyocardially under direct visualization at 5 to 10 points in the central and border areas of all infarcted regions after bypass surgery and before chest closure.

### Outcomes

The primary end point was the safety of the homologous, allogeneic hUC-MSCs and collagen scaffolds, as assessed by the incidence of serious adverse events up to 12 months after surgery, defined as a composite of all-cause death, postoperation myocardial infarction, new tumors, sustained ventricular tachycardia, systemic infection, stroke, allergic reaction, hospitalization because of heart failure, cardiac perforation, pericardial tamponade, ischemia, anaphylaxis, hemodynamic instability, or sustained ventricular arrhythmias (>30 seconds or causing hemodynamic compromise). Additional safety assessments included the clinical monitoring of adverse events, changes in vital signs, electrocardiogram results, and laboratory values (C-reactive protein, creatine kinase–myocardial band, hematology, chemistry, and urinalysis).

The secondary end point was the efficacy of hUC-MSCs and collagen scaffold as assessed according to the cardiovascular magnetic resonance imaging (CMR)–based LVEF and infarct size at 3, 6, and 12 months after treatment. The additional exploratory secondary efficacy end points were based on CMR (indices of ventricular remodeling and function) and clinical assessments (the New York Heart Association class and the Minnesota Living with Heart Failure Questionnaire [total scores range from 0 to 105, with higher scores indicating worse health status]) at 3, 6, and 12 months after treatment. New York Heart Association class was assessed by clinical doctors who are blinded to the whole study.

Other detailed materials and methods (end points, patients, cell and collagen preparation, randomization and masking, and CMR) are reported in the supplementary materials (eMethods in Supplement 2).

### Statistical Analysis

In this prespecified study, all patients with available data were included in analyses. Baseline comparisons were conducted in all patients included in the analysis.

Power calculation of the sample size was performed according to previous studies.^13,14,15,16,17^ Previous studies assumed an average infarct size of total left ventricular mass between 15% and 25% and an expected treatment effect reduction of 4 to 10 absolute percentage points. The SD of myocardial infarct size is 8% to 10%. With a risk of type I error of 5% and type II error of 20%, we wanted to find a reduction in infarct size of 9 absolute percentage points. Assuming an SD of 8, we needed 14 patients in each group. Considering the possible dropout, the total sample size was determined to be 50 patients for 3 groups.

All values were expressed as mean (SD) unless otherwise stated. Intergroup comparison was assessed by repeated-measures analysis of variance with Bonferroni correction or Kruskal-Wallis test. Intragroup differences were compared with paired *t* tests or Mann-Whitney test. A value of *P* < .05 was considered statistically significant. SPSS version 22 (IBM) was used to conduct statistical tests.

## Results

### Patients

**Figure 1.**
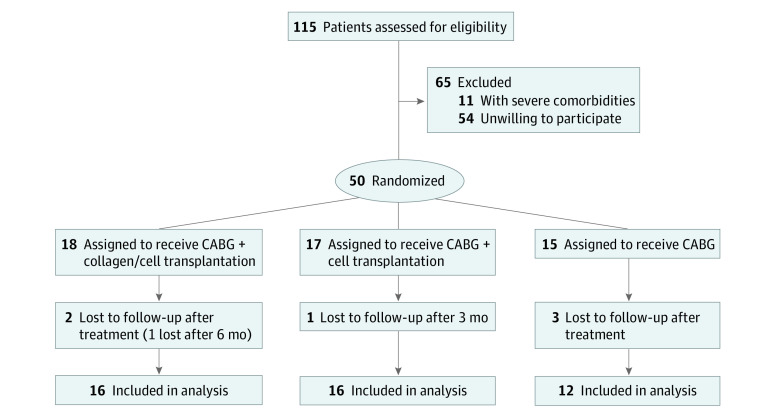
Trial Profile CABG indicates coronary artery bypass grafting.

Figure 1 summarizes the numbers of patients screened, enrolled, and excluded. Of 115 patients assessed for eligibility, 65 were excluded for severe comorbidities or unwillingness to participate (all inclusion and exclusion criteria are listed in eTables 4 and 5 in Supplement 2). A total of 50 patients (mean [SD] age, 62.6 [8.3] years; 38 men [76%] and 12 women [24%]) were enrolled, of whom 18 were randomized to the collagen/cell group, 17 to the cell group, and 15 to the control group. There were no significant differences among the 3 groups of patients in baseline characteristics (Table 1) and perioperative data (eTable 2 in Supplement 2). Although there was an imbalance of more patients with anterior infarction in the cell group (7 patients [43.8%]) than in the collagen/cell group (3 patients [18.8%]) and the control group (2 patients [16.7%]), the infarct artery and bypass vessel did not differ among 3 groups (Table 1; eTable 2 in Supplement 2). All patients received a left internal mammary artery graft and at least 3 vessel grafts. The cell- or collagen/cell-treated cardiac areas were concomitantly revascularized. Routine medication and management were used for all 3 groups of patients perioperatively and after initial hospitalization. Briefly, β-blockers, nitrate, statins, aspirin, and ticagrelor were used. The last patient completed 12 months of follow-up in August 2019.

**Table 1.** Baseline Characteristics (Safety Analysis Set)

Characteristic^a^	No. (%)^b^		
	Collagen/hUC-MSCs (n = 16)	hUC-MSCs (n = 16)	Control (n = 12)
Demographic characteristics and medical history			
Male sex	13 (81.30)	12 (75.00)	7 (58.30)
Preoperation hospital days, median (IQR)	9.00 (7.00-13.00)	9.00 (7.00-16.25)	9.00 (6.00-14.50)
Total hospital days, median (IQR)	25.00 (22.25-26.75)	29.50 (22.50-36.58)	27.00 (21.00-31.25)
Age, mean (SD), y	59.6 (7.9)	63.6 (8.6)	65.2 (7.9)
Hypertension	10 (62.5)	14 (87.5)	9 (75.0)
Hyperlipidemia	2 (12.5)	1 (6.3)	1 (8.3)
Diabetes	8 (50.0)	4 (25.0)	8 (66.7)
Tobacco use	4 (25.0)	7 (43.8)	3 (25.0)
Alcohol use	1 (6.3)	4 (25.0)	3 (25.0)
Previous PCI	2 (12.5)	3 (18.8)	0
Stroke	2 (12.5)	3 (18.8)	2 (16.7)
Peripheral artery disease	0	1 (6.30)	1 (8.30)
Hepatic disease	1 (6.30)	2 (12.50)	0
Physical measurements			
Body temperature, median (IQR), °C	36.60 (36.20-36.80)	36.50 (36.50-36.58)	36.50 (36.3-36.78)
Heart rate, median (IQR), bpm	82.00 (73.25-85.50)	78.50 (67-80.75)	74.50 (65.75-78.00)
Breaths/min, median (IQR)	20 (18.75-20.00)	20 (18.00-20.00)	20 (18.25-20.00)
Systolic blood pressure, mean (SD), mm Hg	125.56 (24.79)	129.13 (12.19)	115.92 (17.16)
Diastolic blood pressure, mean (SD), mm Hg	80.69 (17.83)	79.63 (11.49)	71.08 (8.04)
Spo_2_(%), median (IQR)	98.00 (97.00-98.00)	97.5 (96.25-98.00)	98 (97.25-99.00)
Body weight, mean (SD), kg	70.81 (11.27)	67.66 (10.55)	63.66 (8.06)
Body height, median (IQR), cm	167 (165.00-170.00)	166.50 (162.75-171.50)	165.00 (158.75-169.50)
Body mass index, mean (SD)^c^	25.52 (3.32)	24.47 (3.38)	23.59 (2.28)
≤30 d From symptom appearance to surgery	3 (18.8)	7 (43.8)	3 (25)
Infarct artery distribution			
Left main artery	15 (93.8)	15 (93.8)	10 (83.3)
Left anterior descending artery	15 (93.8)	15 (93.8)	10 (83.3)
Left circumflex artery	15 (93.8)	15 (93.8)	8 (66.7)
Right coronary artery	13 (81.3)	15 (93.8)	10 (83.3)
No. of vessels >50%			
None	1 (6.3)	1 (6.3)	0
1	0	0	1 (8.3)
2	1 (6.3)	0	0
3	7 (43.8)	6 (37.5)	2 (16.7)
>3	7 (43.8)	9 (56.3)	9 (75.0)
Prior infarction area			
Apex	2 (12.5)	6 (37.5)	6 (50)
Anterior	3 (18.8)	7 (43.8)	2 (16.7)
Free wall	1 (6.3)	4 (25)	4 (33.3)
Posterior	0	1 (6.3)	0
Inferior	2 (12.5)	5 (31.3)	2 (16.7)
Septal	3 (18.8)	3 (18.8)	6 (50)
Medication before initial hospitalization			
Aspirin	5 (31.3)	9 (56.3)	5 (41.7)
Clopidogrel	5 (31.3)	6 (37.5)	2 (16.7)
Statin	3 (18.8)	8 (50.0)	5 (41.7)
β-blocker	3 (18.8)	7 (43.8)	2 (16.7)
ACEI or ARB	3 (18.8)	4 (25.0)	4 (33.3)
Nitrate	4 (25.0)	2 (12.5)	2 (16.7)
Calcium channel blockers	1 (6.3)	1 (6.3)	1 (8.3)
Complication			
Hydrothorax	1 (6.3)	2 (12.5)	0
Pulmonary infection	2 (12.5)	1 (6.3)	0
Hydropericardium	1 (6.3)	1 (6.3)	0
Seroperitoneum	0	1 (6.3)	0
Cardiac function			
LVEF (3-D echocardiogram) below average^d^	10 (62.5)	8 (50.0)	6 (50.0)
LVEF (CMR) below average^e^	10 (62.5)	8 (50.0)	7 (58.3)
NYHA heart function class			
Class III	4 (25)	8 (50)	7 (58.3)
Class IV	12 (75.0)	8 (50.0)	5 (41.7)
Myocardial damage marker			
Abnormal increased CK-MB	3 (18.8)	2 (12.5)	2 (16.7)
Abnormal increased cTnT	5 (31.3)	2 (12.5)	2 (16.7)

Abbreviations: ACEI, angiotensin-converting enzyme inhibitor; ARB, angiotensin receptor blocker; bpm, beats per minute; CK-MB, creatine kinase–myocardial band; CMR, cardiac magnetic resonance imaging; cTnT, cardiac troponin T; hUC-MSC, human umbilical cord–derived mesenchymal stromal cell; IQR, interquartile range; LVEF, left ventricular ejection fraction; NYHA, New York Heart Association; PCI, percutaneous coronary intervention.

^a^ All vital signs were obtained once patients were admitted to the cardiothoracic surgery department. Results from the first examination after admission to the hospital before surgery were set as the baseline.

^b^ All values are presented as number and percentage unless otherwise noted. Medians and IQRs are given for values that are not equally distributed.

^c^ Calculated as weight in kilograms divided by height in meters squared.

^d^ The mean value of LVEF for all groups assessed by 3-D echocardiogram before surgery was 35.56%.

^e^ The average LVEF assessed by CMR before surgery was 31.32%.

### Cells and Collagen

The clinical-grade hUC-MSCs were shown to be positive for mesenchymal stromal cell markers (CD73, CD90, and CD105) and negative for other markers (CD14, CD19, CD34, CD45, and HLA-DR) by flow cytometry analysis (eFigure 1A in Supplement 2). They were spindle-shaped cells (eFigure 1B in Supplement 2) and had great potential for bone, adipocyte, and cartilage differentiation (eFigure 1C-E in Supplement 2).

**Figure 2.**
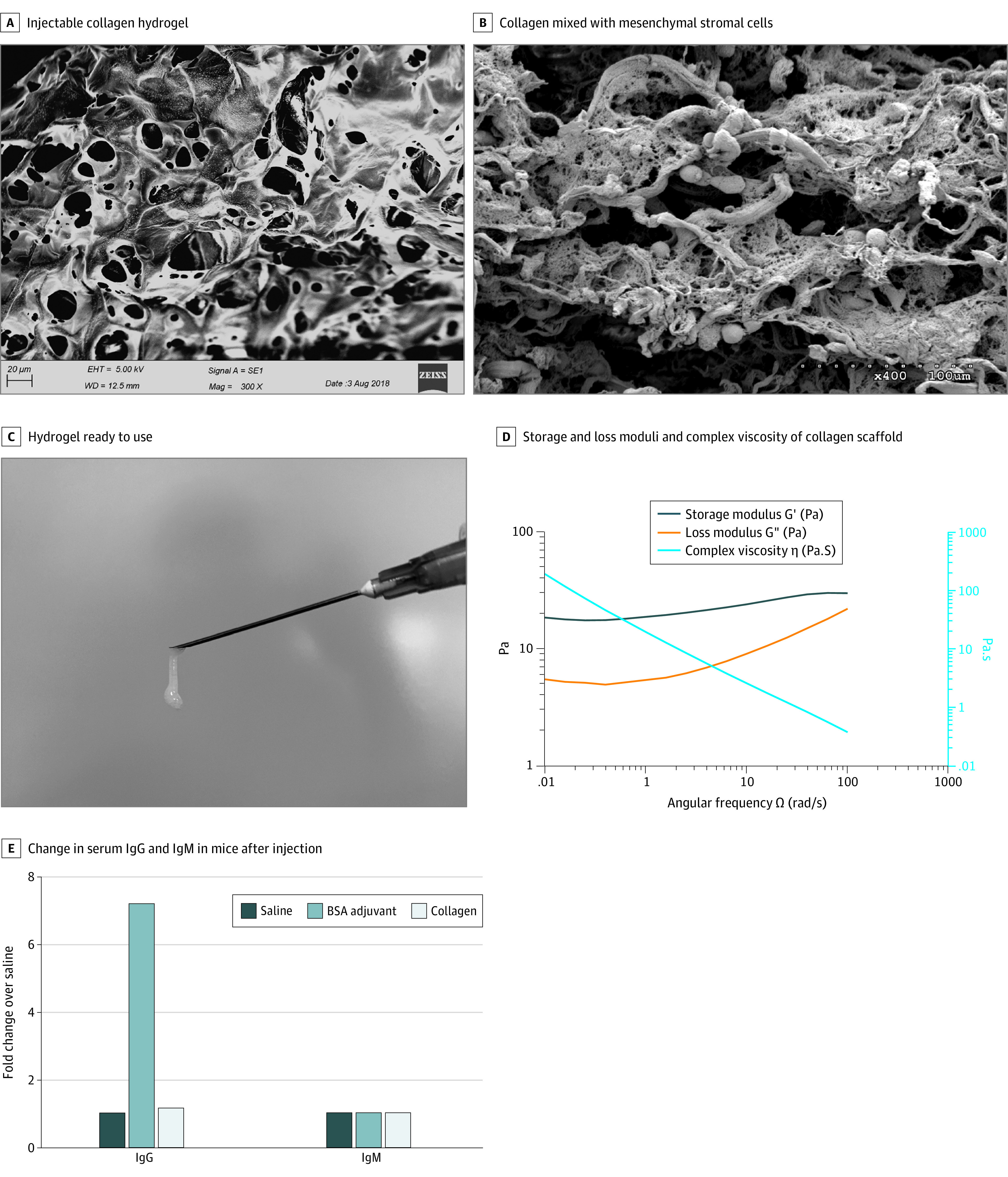
Characterization of Hydrogel and hUC-MSCs A, Scanning electron microscopic image of the bovine-derived injectable collagen hydrogel displaying fibrillar networks of collagen fibers suitable for cell attachment. Scale bar: 20 μm. B, Electron microscopic image of the collagen mixed with mesenchymal stromal cells. Scale bar: 100 μm. C, Injectable collagen hydrogel ready to use. D, Storage (G′) modulus, loss (G″) modulus, and complex viscosity of the collagen scaffold mixed with cells. E, Change in serum IgG and IgM of mice after collagen hydrogel injection. Bovine serum albumin (BSA) adjuvant was used as a positive control. Saline was used as a negative control. The changes in IgG and IgM were similar between saline and collagen hydrogel. hUC-MSC indicates human umbilical cord–derived mesenchymal stromal cell.

The injectable porous collagen scaffold was a white viscous gel that could be injected with a 27G needle, and scanning electron microscope analysis indicated that it was composed of collagen fibers (Figure 2A-C). The rheological characteristics of the collagen scaffolds mixed with cells were analyzed. The oscillatory frequency sweeps of the collagen scaffold performed showed formation of structured, solid-like hydrogel after mixing with cells (Figure 2D). Figure 2B is the scanning electron microscope image of the combined product of collagen and cells. Biological safety of the collagen scaffold was evaluated before application, and it was shown to meet the Chinese Criterion for Medical Devices GB16886 regarding the absence of allergens and biological toxicity (Figure 2E; eMethods, eFigures 4 and 6, and eTable 3 in Supplement 2).

### Primary End Points

All 50 patients received the full intended treatment of hUC-MSCs plus CABG, collagen/cells plus CABG, or CABG alone. No significant differences were observed among groups for the relative incidences of adverse events and serious adverse events (Table 2) within 12 months after treatment administration. Treatment with collagen and/or stromal cells did not increase the frequency of arrhythmia. One patient in the collagen/cell group was hospitalized 1 year after treatment because of insufficient water intake restriction (leading to chest distress) and recovered soon after that hospitalization. One patient in the cell group was hospitalized with an upper respiratory tract infection. No serious adverse events were seen in the control group.

**Table 2.** Serious Adverse Events

Adverse event	No. (%)		
	Collagen/hUC-MSCs (n = 15)	hUC-MSCs (n = 15)	Control (n = 12)
All-cause death	0	0	0
New tumor	0	0	0
Sustained ventricular tachycardia^a^	0	0	0
Systemic infection	0	0	0
Stroke	0	0	0
Allergic reaction	0	0	0
Hospitalization because of heart failure^b^	1 (6.7)	1 (6.7)	0
Severe arrhythmia needing intervention	0	0	0

Abbreviation: hUC-MSC, human umbilical cord–derived mesenchymal stromal cell.

^a^ Sustained ventricular tachycardia was defined as ventricular tachycardia for greater than 30 seconds or requiring termination in less than 30 seconds due to hemodynamic compromise.

^b^ *P* = .68.

Laboratory results indicated that there were no significant differences in immunoglobulin, myocardial damage markers, or renal or liver function among all groups after surgery (eFigure 2 in Supplement 2). The alexin C3 level in control group patients was significantly higher than in cell group patients at 1 week after surgery, even though all distributions were in the normal range (eFigure 2A in Supplement 2). Myocardial biomarkers (creatine kinase–myocardial band) showed substantial increases in a few patients but did not significantly differ among the groups (eFigure 2F in Supplement 2). Overall, no increased systematic inflammation or other immune response was observed in either of the treatment groups, which indicates the safety of collagen and/or cell application.

### Secondary End Points

A total of 42 patients completed 12 months of follow-up. One patient had no cardiac scar tissue (collagen/cell group). Four patients (2 in the cell group and 2 in the control group) at baseline and 1 patient (control group) at 12 months lacked scar size data owing to intolerance of delayed contrast-enhanced CMR for infarct assessment, which required an extra half-hour of exposure. Thus, there were 36 patients (14 in the collagen/cell group, 13 in the cell group, and 9 in the control group) with scar size data analyzed at 12 months.

**Figure 3.**
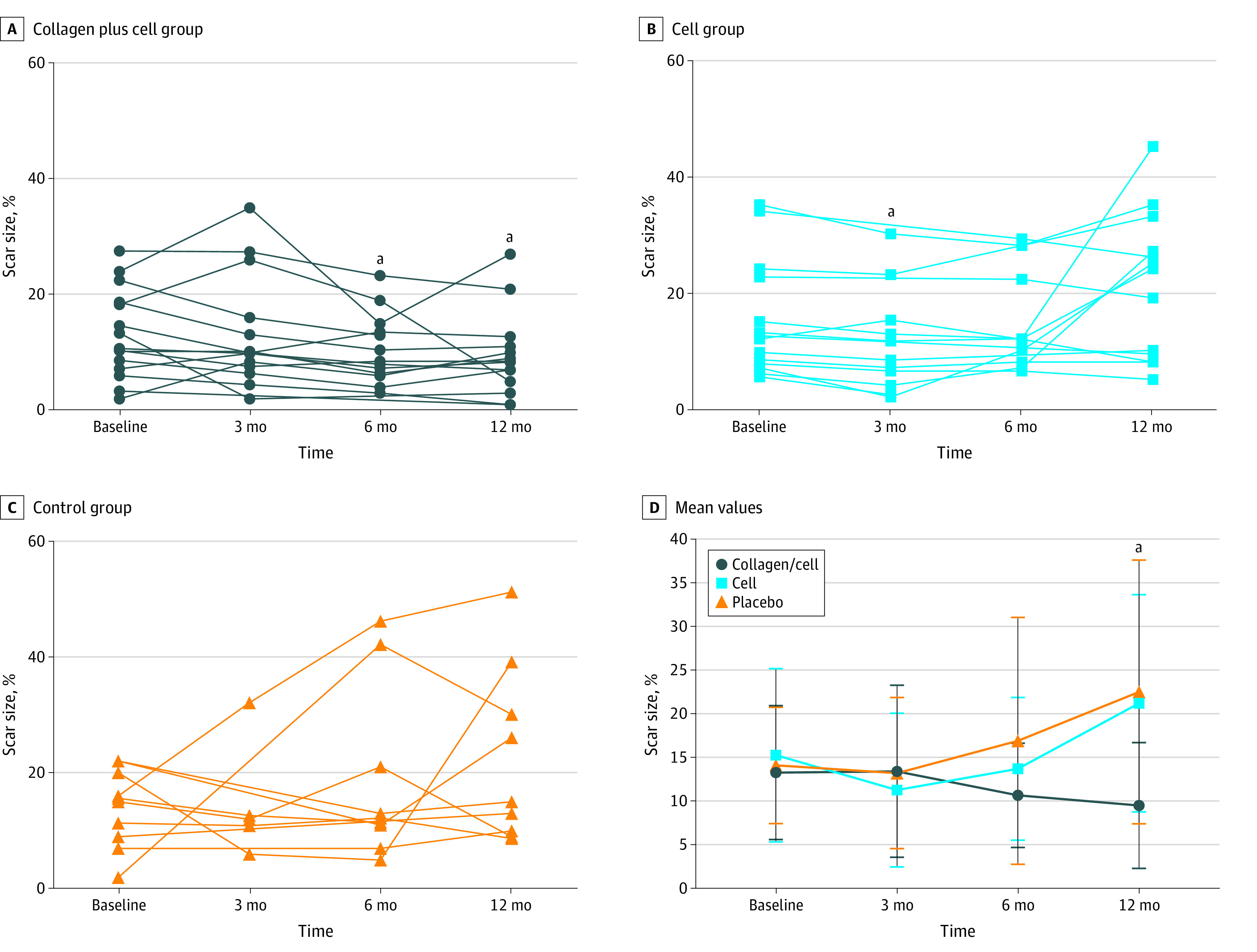
Myocardial Infarction Scar Size Measured by CMR Change in scar size shown for the collagen/cell group (A), cell group (B), and control group (C). Panel D shows the mean (SD) values. CMR indicates cardiovascular magnetic resonance imaging. ^a^*P* = .049, calculated by analysis of variance or Kruskal-Wallis test, post hoc multiple comparisons with Bonferroni correction.

CMR parameters and life quality assessments are reported in eTable 1 in Supplement 2. Changes in left ventricular transmurality (eFigure 5 in Supplement 2) and mean infarct size as a percentage of the left ventricular mass (Figure 3) decreased after treatment in the collagen/cell group but increased in the cell group and control group (Figure 3; eTable 1 in Supplement 2). At 12 months after treatment, the mean infarct size percentage change was −3.10% (95% CI, −6.20% to −0.02%; *P* = .05) in the collagen/cell group, 5.19% (−1.85% to 12.22%, *P* = .35) in the cell group, and 8.59% (−3.06% to 20.25%, *P* = .21) in the control group.

The CMR LVEF increased in all group patients (eFigure 3 in Supplement 2). When we focused on patients with baseline LVEF of 40% of less (15 in the collagen/cell group, 12 in the cell group, and 10 in the control group), mean LVEF in the collagen/cell group patients increased by 9.14% (95% CI, 1.19% to 17.10%; *P* = .03), 9.84% (95% CI, 1.20% to 18.49%; *P* = .03), and 9.35% (95% CI, 1.96% to 16.75%; *P* = .02) at 3, 6, and 12 months, respectively, while that in the cell group increased by 3.38% (95% CI, −2.06% to 8.83%; *P* = .19), 3.39% (95% CI, −2.05% to 9.98%; *P* = .17), and 6.59% (95% CI, 2.61% to 10.56%; *P* = .004), respectively. The control group showed mean increases of 4.71% (95% CI, −4.39% to 13.82%; *P* = .24), 4.40% (95% CI, −1.84% to 10.64%; *P* = .14), and 3.62% (95% CI, −3.25% to 10.50%; *P* = .25).

The health-related quality of life of patients was assessed by the Minnesota Living with Heart Failure Questionnaire (total scores range from 0 to 105, with higher scores indicating worse health status). The mean change in score was −22.60 (95% CI, −40.18 to −5.02; *P* = .02) in the collagen/cell group, −24.20 (95% CI, −34.82 to −13.58; *P* < .001) in the cell group, and −16.17 (95% CI, –26.31 to −6.02; *P* = .005) in the control group at 12 months.

The proportion of patients with New York Heart Association heart function class improvement increased for all groups after treatment, with no significant differences at any time point (eFigure 2H in Supplement 2). The New York Heart Association heart function class improved at 12 months compared with baseline in 13 patients (86.7%) in the collagen/cell group, 11 (73.3%) in the cell group, and 8 (66.7%) in the control group (eFigure 2 and eTable 1 in Supplement 2).

## Discussion

We performed the first clinical trial to our knowledge to explore the safety and effect of injectable hydrogel loaded with mesenchymal stromal cells. This study suggests that intramyocardial injection of collagen hydrogel laden with hUC-MSCs during CABG is safe and feasible for the treatment of patients with myocardial infarction. In this study, bovine collagen was adopted as a cell delivery vehicle, and no significant difference in adverse response was observed among the treatment groups and control group. In clinical trials in which biomaterials seeded with cells were epicardially delivered during CABG, cardiac function increase^18^ and nonvariable segment recuperation^19,20^ were observed. In the present study, patients had improved cardiac function (measured by LVEF and New York Heart Association heart function class), viable cardiac tissue size and quality of life (measured by Minnesota Living with Heart Failure Questionnaire), and decreased left ventricular end-diastolic volume and left ventricular end-systolic volume (eTable 1 in Supplement 2). Although there were more patients with anterior infarction in the cell group than in the collagen/cell and control groups, the infarct artery and bypass vessel did not differ among the 3 groups (Table 1; eTable 2 in Supplement 2). Scar size remained stable in the collagen/cell group but increased in the cell group and control group at 12 months (eTable 1 in Supplement 2). Previous studies^2,21,22^ similarly reported that injection of autologous regenerative cells during CABG did not reduce scar size, and even greater scar transmurality was observed in CABG alone and with intramuscular injection. Because there were large differences in the assessment criteria and patient characteristics in previous studies, it is difficult to compare the effects of cell therapy, biomaterial patches, and hydrogels among different studies.^22,23,24^ According to a meta-analysis,^2,25^ clinical trials of cell therapy for cardiac regeneration in heart failure have usually yielded neutral or, at most, marginally positive results.

### Limitations

This study has some limitations. First, whether the enhanced benefit of the hydrogel group was derived from the remaining transplanted cells or the collagen hydrogel itself is still unknown,^26,27^ because there was no collagen-only group. Second, cell transplantation was performed by a cardiac surgeon, which means that it was difficult to blind the surgeons, because there is a different resistance force between single-cell injection and collagen-cell mixture injection. We kept the patients, clinical doctors, and echocardiography and CMR investigators blinded to the trial group assignments to reduce possible bias. Third, owing to the small sample size of a single-center trial, it is not realistically possible to provide adjustments of safety in the context of additional risk factors or covariates. This is a limitation here but also an important consideration for later work.

## Conclusion

To the best of our knowledge, this study is the first clinical trial to evaluate an injectable cell-laden biomaterial for cardiac repair. Although it is limited by a small sample size, this study provides the basis for larger trials to evaluate the safety and efficacy of cell-laden collagen gel therapy.

## Notes

Supplement 1. **Trial Protocol**

Supplement 2. **eMethods.** Materials and Methods **eFigure 1.** Characterization of hUC-MSCs **eFigure 2.** Laboratory Results of Safety Assessment **eFigure 3.** Restoration of Cardiac Function and Attenuation of Negative LV Remodeling **eFigure 4.** Blood Cell Count and Body Weight Change **eFigure 5.** Representative Cardiac Magnetic Resonance Images **eFigure 6.** Analysis of Immune Cells in Mouse Spleen After Collagen Hydrogel Analysis **eTable 1.** CMR and MLHFQ Data (Safety Analysis Set) **eTable 2.** Parameters of CABG Operation **eTable 3.** Intradermal Irritation Assay **eTable 4.** Inclusion Criteria **eTable 5.** Exclusion Criteria

Supplement 3. **Data Sharing Statement**
