# Supplementary material for: Effect of Intramyocardial Grafting Collagen Scaffold With Mesenchymal Stromal Cells in Patients With Chronic Ischemic Heart Disease: A Randomized Clinical Trial
Source: JAMA Netw Open. 2020 Sep 10;3(9):e2016236. doi: 10.1001/jamanetworkopen.2020.16236 (PMC7489863; doi:10.1001/jamanetworkopen.2020.16236)
Supplement: Supplement 1. — Trial Protocol [file jamanetwopen-e2016236-s001.pdf]

Human Umbilical Cord-derived Mesenchymal Stromal Cells With Injectable Collagen Scaffold Transplantation for Chronic Ischemic Cardiomyopathy

IRB Protocol Number:2015-106-01

Principal Investigator: Dongjin Wang, MD, PhD, Department of Thoracic and Cardiovascular Surgery, the Affiliated Drum Tower Hospital of Nanjing University Medical School

Draft or Version Number: 2.0

30 September 2015

Funding Acknowledgements

Research reported in this publication was supported by Strategic Priority Research Program of the Chinese Academy of Sciences [XDA16040000]; the Key Research Program of the Chinese Academy of Sciences [ZDRW-ZS-2016-2-2]; the National Key R&D Program of China [2016YFC1000800, 2016YFC1000808]; the National Natural Science Foundation of China [81370239]; the Key Project supported by Medical Science and Technology Development Foundation, Nanjing Department of Health [201605016]; the Key Project supported by Nanjing Medical Science and technique Development Foundation [QRX17044]; and the Youth Innovation Promotion Association CAS Project [2016096]. The content is solely the responsibility of the authors and does not necessarily represent the official views of the above mentioned organization.

Statement of Compliance

The study will be conducted in accordance with the International Conference on Harmonisation guidelines for Good Clinical Practice (ICH E6), the Code of Federal Regulations on the Protection of Human Subjects (45 CFR Part 46), and PCORI Terms of Award. All personnel involved in the conduct of this study have completed human subject protection training and Good Clinical Practice (GCP) training.

Signature Page

The signature below constitutes the approval of this protocol and the attachments and provides the necessary assurances that this trial will be conducted according to all stipulations of the protocol, including all statements regarding confidentiality, and according to local legal and regulatory requirements and applicable US federal regulations and ICH guidelines.

To be signed by all of the following:

Study Principal Investigator: Jianwu Dai

Clinical site Principal Investigator: Donjin Wang

Protocol Version 2.0

30Sep2015

I have read and understand the information in this Study Protocol and will ensure that all associates, colleagues, and employees assisting in the conduct of the study are informed about the obligations incurred by their contribution to the study.

Signed: 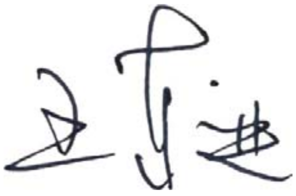

Name: Donjin Wang

Title: MD & PhD

Site/Location: Department of Thoracic and Cardiovascular Surgery, the Affiliated Drum Tower Hospital of Nanjing University Medical School

Study Role: Principal Investigator

Table of Contents

Funding Acknowledgements....2
Statement of Compliance....3
Signature Page....4
Table of Contents....5
List of Abbreviations....8
Protocol Summary....10
1 Schematic of Study Design....11
2 Key Roles and Contact Information....12
3 Introduction: Background information and scientific rationale....13
3.1 Background Information....13
3.2 Hypotheses....14
3.3 Potential Risks and Benefits....15
4 Objectives....15
4.1 Study Objectives....15
4.2 Study Outcome Measures....15
5 Study Design....16
6 Study Enrollment and Withdrawal....16
6.1 Subject Inclusion Criteria....17
6.2 Subject Exclusion Criteria....17
6.3 Strategies for Recruitment and Retention....18
6.4 Treatment Assignment Procedures....19
6.5 Subject Withdrawal....19
6.6 Premature Termination or Suspension of Study....20
7 Baseline assessment....20
8 Study Intervention....21
8.1 Study Procedural Interventions Description....21

8.2 Procedures for Training of Clinicians on Procedural Intervention 22
9 Follow-Up 22
10 Study closure 23
10.1 End of Study 23
10.2 Early discontinuation of study 23
11 Assessment of Safety 23
11.1 Specification of Safety Parameters 23
11.2 Time Period and Frequency for Event ASSESSMENT AND Follow-Up 24
11.3 Characteristics of an Adverse Event 24
11.4 Reporting Procedures 25
11.5 Halting Rules 26
12 Study Oversight 26
12.1 Study management Group (SMG) 27
13 Clinical Site Monitoring 27
13.1 Screening/Enrollment Monitoring Reports 27
13.2 Site Monitoring Calls 27
13.3 Site Visits 27
14 Statistical Considerations 28
14.1 Study Hypotheses 28
14.2 Sample Size Considerations 28
15 Source Documents & Access to Source Data/Documents 28
15.1 Source Documents 28
15.2 Maintenance of and Access to Source Documents 29
16 Quality Control and Quality Assurance 29
16.1 Electronic Data Capture 29
16.2 Data Entry 29
17 Ethics/Protection of Human Subjects 30
17.1 Ethical Standard 30
17.2 Ethical approval 30
17.3 Informed Consent Process 30

17.4 Subject Confidentiality and data protection 30

18 Literature References 31

List of Abbreviations

AE	Adverse event
ADL	Activities of daily living
CABG	Coronary artery bypass graft
CIHD	Chronic ischemic heart disease
CM	Cardiomyocytes
CMR	Cardiac magnetic resonance
CRF	Case report forms
CRP	C-reactive protein
CVD	Cardiovascular disease
ECG	Electrocardiogram
ECM	Extracellular matrix
GCP	Good clinical practice
HF	Heart failure
ICF	Informed Consent Form
ICH	International Conference on Harmonisation
ICMJE	International committee of medical journal editors
iPSC	induced pluripotent stem cells
IRB	Institutional review board
LM	Left main artery
LV	Left ventricular
LVEF	Left ventricular ejection fraction
MD	Medical doctor/physician
MI	Myocardial infarction
MLHFQ	Minnesota Living with Heart Failure Questionnaire
MRI	Magnetic resonance imaging

N	Number (typically refers to subjects)
NIH	National institutes of health
NSTEMI	Non-ST-segment elevation myocardial infarction
NYHA	the New York Heart Association
PHI	Protected health information
PI	Principal investigator
QA	Quality assurance
QC	Quality control
RA	Research assistant/research coordinator
SAE	Serious adverse event
SD	standard deviation
SMG	Study management Group
SP	Standard operating procedure
STEMI	ST-segment elevation myocardial infarction
WHO	World health organization

Protocol Summary

Title	Human Umbilical Cord-derived Mesenchymal Stromal Cells With Injectable Collagen Scaffold Transplantation for Chronic Ischemic Cardiomyopathy
Precis	This is a randomized controlled trial in patients with CIHD patients. The study will recruit 50 patients randomized to CABG plus cell and/or collagen gel versus regular CABG operation. The primary study and analysis will compare safety at one (1) year follow-up.
Objectives	To determine whether cell laden hydrogel treatment is safe and feasible, and the efficacy at 12 months of follow-up.<br>Primary Outcome: SAE<br>Secondary: CMR-based LVEF and infarct size
Population	CHID patients with left ventricular ejection fraction (LVEF) ≤ 45% (assessed by 3D echocardiography) and who needed CABG and not suitable for percutaneous coronary intervention revascularization.
Sample Size	50 subjects
Phase	I
Number of Sites	1
Description of Intervention	Patients were assigned to collagen/cell group, cell group and control group and received hUC-MSCs (1×10^8/1.5 ml PBS) plus collagen scaffold (1 ml) and CABG, hUC-MSCs (1×10^8/2.5 ml PBS) and CABG, or CABG alone, respectively. Cells and/or hydrogel were injected intra-myocardial, under direct visualization, at 5-10 points in the central and border areas of all infarcted region after bypass operation and before chest closure.
Study Duration	Approximately 3.5 years
Estimated Time to Complete Enrollment	Approximately 2.5 years

1 Schematic of Study Design

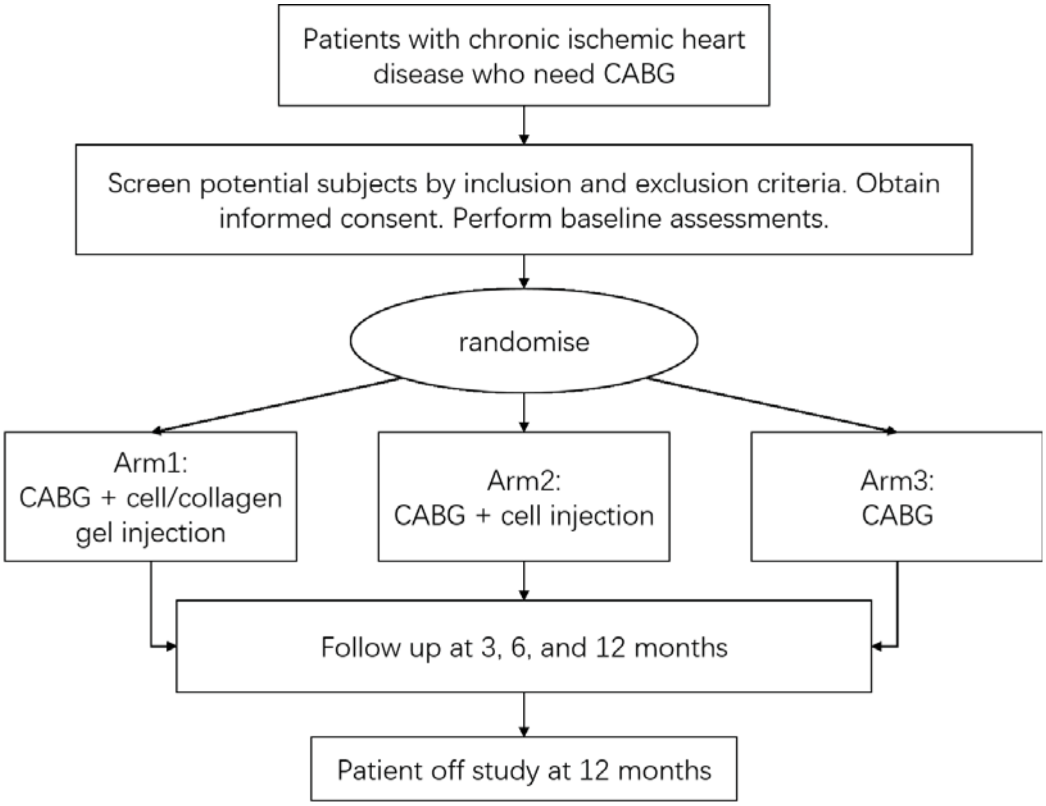

2 Key Roles and Contact Information

Study Principal Invistigator	Dr. Jianwu Dai
Address	Key Laboratory of Molecular Developmental Biology, Institute of Genetics and Developmental Biology, Chinese Academy of Sciences, Beijing, China
Executive study coordinator	Dr. Yannan Zhao
Address	Key Laboratory of Molecular Developmental Biology, Institute of Genetics and Developmental Biology, Chinese Academy of Sciences, Beijing, China
Clinical site Principal Investigator	Dr. Dongjin Wang
Address	Department of Thoracic and Cardiovascular Surgery, the Affiliated Drum Tower Hospital of Nanjing University Medical School, 321 Zhongshan Road, Nanjing, 210008, China
Study management group	
Name	Job title
Qiang Wang	Recruiting physician
Xiaojun He	Recruiting physician
He Zhang	Recruiting physician

3 Introduction: Background information and scientific rationale

3.1 Background Information

An estimated 17 million deaths occur due to cardiovascular disease (CVD) per year, as the leading cause of global death. Heart disease is also the leading cause of registered death around the world. As population aging and treatment continue to improve, patients are more likely to survive initial cardiac events such as myocardial infarctions. For chronic ischemic heart disease (CIHD) patients with left main artery (LM) or three-vessel disease, particularly when the proximal LAD coronary artery was involved, the benefit of mortality reduction from coronary artery bypass graft (CABG) over a strategy of initial medical therapy was established in a meta-analysis of seven RCTs[1] more than two decades ago, and has been corroborated in more recent studies[2, 3]. This contributing to a rise in the prevalence of heart failure (HF). There was an increased incidence of post-infarction chronic heart failure (HF), even among those treated with evidence-based therapies, such as angiotensin-converting enzyme inhibitors, β-blockers, mineralocorticoid receptor antagonists, and angiotensin receptor neprilysin inhibitors. The treatments for CVD are limited because the heart is a terminally differentiated organ with little regenerative capacity[4], which means it is unable to adequately compensate for the overwhelming cardiomyocyte loss that underlies the development of heart failure. Heart transplantation is the only successful treatment for end-stage heart failure but is limited by the shortage of donor hearts.

Over the past 2 decades, there has been extensive interest[5] in using stem cells to treat patients with myocardial infarction (MI) and ischemic heart failure via hypothetical mechanisms involving the generation of new myocardial tissue intended to replace myocardium that has been irreversibly lost[6, 7] or to release molecules[8] that harness endogenous repair mechanisms. Stem cells are defined by their ability to self-renew and differentiate into mature cell type. Many types of cells, such as skeletal myoblasts[9, 10], bone marrow-derived cells[6], unselected bone marrow-derived cells[11, 12], purified hematopoietic stem and progenitor cells[13, 14], cardiac-committed cells[15-17], induced pluripotent stem cells (iPSCs) and mesenchymal stem cells[18]have been adopted for cardiac repair and regeneration. According to these studies, the application of mesenchymal stem cells holds great promise owing to the strong paracrine (angiogenic, anti-inflammatory and immunomodulatory) effects and lack of ethical issues.

Cells can be delivered through numerous methods, such as intramyocardial injection, intracoronary injection and retrograde coronary venous injection, to cardiac tissue at different stages of heart disease. However, the cell delivery techniques used thus far have all been fraught with poor efficiency. The cells delivered into cardiac tissues have rapidly diffused via the venous system within a few heartbeats, even though intramyocardial injections yield higher transfer rates than intracoronary and retrograde coronary venous injection[19]. This primarily leads to poor cell engraftment, retention, and survival of transplanted cells in heart

tissue.

The use of hydrogels may be beneficial for solving this problem[20-27]. Hydrogels are biomaterials designed to physically support delivered cells, to maintain their placement in the injury zone. Collagen is the predominant protein in mammalian extracellular matrix (ECM), and it provides structural support for maintaining tissue integrity and contributes to the specificity of ECM microenvironments[28]. In myocardium, clusters of cardiomyocytes (CMs) are encircled by collagen, as the most plentiful protein. Collagen has great advantages as a biomaterial resource for cell transplantation therapy due to its good biocompatibility, porosity, biodegradability and low immunity. In recent decades, animal studies have indicated that collagen scaffolds can benefit cardiac regenerative therapy[29]. Collagen-based biomaterials have already been used as stem cell delivery vehicles to enhance cell adhesion, retention, and engraftment[21, 22]. Most studies of stem cells with injectable hydrogels have been implemented in small animal models, and such applications have not been examined in clinical studies.

Here, we developed an injectable porous collagen scaffold hydrogel derived from bovine collagen tissue. This material was administered intramyocardially into border zone heart tissue, enabling better cell retention, cardiac function restoration and myocardial infarction prevention. Our previous preliminary experiment demonstrated that the hydrogel was safe and promoted post-MI cardiac function with a decrease in the scar tissue size percentage and, an increase in transplanted cell retention and neo-angiogenesis in the scar area in pigs. This motivated the testing of collagen hydrogel in post-MI patients. Accordingly, we developed a randomized, single center trial to evaluate the safety, feasibility and preliminary efficacy of the intramyocardial delivery of collagen hydrogel with clinic-grade hUC-MSCs in post MI patients with left ventricular (LV) dysfunction right after CABG. This injectable biomaterial approach represents an alternative to cell-based regenerative strategies for the heart.

3.2 Hypotheses

Our hypotheses for the primary analysis at year one follow-up are:

Hypothesis 1: The treatment of cell laden hydrogel injection is safe and feasible, and the efficacy at 12 months of follow-up.

Hypothesis 2: The treatment of CABG plus cell laden hydrogel will lead to smaller infarct size percentage at 12 months than the regular CABG.

Hypothesis 3: The treatment of CABG plus cell laden hydrogel will lead to higher LVEF at 12 months at 12 months than the regular CABG.

3.3 Potential Risks and Benefits

3.3.1Potential Risks

Potential risks include:

- fatigue due to answering study questionnaires
- anaphylactic reaction, immune rejection response; increased systemic inflammation.
- new myocardial infarction, new tumors, stroke, cardiac perforation, sustained ventricular arrhythmias.

The medical risks of participating in this study are those inherent to assigned treatments which are standards of care.

systemic inflammation, tissue injury because of normal operation, hemodynamic instability, post-operative bleeding or infection, post-operative abnormal mental condition, blood clot, organ abnormality because of surgical trauma and fluid management, complications related to anesthesia, and, in rare cases, death.

In addition, there may be risks associated with the study that are unknown or unexpected.

3.3.2Potential Benefits

There will be higher LVEF, smaller scar size percentage, and better life quality for patients.

4 Objectives

4.1 Study Objectives

To determine whether cell laden hydrogel treatment is safe and feasible, and the efficacy at 12 months of follow-up.

4.2 Study Outcome Measures

4.2.1Primary Outcome

The primary endpoint was the safety of the homologous, allogeneic hUC-MSCs and collagen scaffolds, as assessed by the incidence of serious adverse events (SAE) [time frame of up to 12 months after surgery], which were defined as a composite of all-cause death, post-operation MI, new tumors, sustained ventricular tachycardia, systemic infection, stroke, allergic reaction, hospitalization because of HF, cardiac perforation, pericardial tamponade,

ischemia, anaphylaxis, hemodynamic instability, or sustained ventricular arrhythmias (>30 s or causing hemodynamic compromise). Additional safety assessments included the clinical monitoring of adverse events (AE), changes in vital signs, electrocardiogram (ECG) and laboratory values (CRP [C-reactive protein], CK-MB, hematology, chemistry, and urinalysis).

4.2.2Secondary Outcome

The secondary endpoint was the efficacy of hUC-MSCs and collagen scaffold, as assessed according to the CMR-based LVEF and infarct size at 3, 6 and 12 months after treatment. The additional exploratory secondary efficacy end points were based on CMR (indices of ventricular remodeling and function) and clinical assessments (the New York Heart Association (NYHA) class and the MLHFQ) at 3, 6 and 12 months after treatment. NYHA class was assessed by clinical doctors who are blinded to the whole study. MLHFQ was completed by patients themselves who are blinded.

5 Study Design

This is an unblinded, single-center, randomized controlled clinical trial of CABG plus cell and or collagen gel for CHID patients with left ventricular ejection fraction (LVEF) ≤ 45% (assessed by 3D echocardiography) and who needed CABG and not suitable for percutaneous coronary intervention revascularization. A sample size of 50 patients will be enrolled with approximately equal numbers of patients randomized to collagen/cell group (CABG plus mesenchymal stromal cell laden collagen gel injection), cell group (CABG plus mesenchymal stromal cell injection), and control group (regular CABG operation). The duration of enrollment is expected to last approximately 2.5 years. Upon enrollment at baseline, patients will fill out study questionnaires, undergo a professional physical examination, regular lab examination, life quality questionnaire, echocardiography and cardiac magnetic resonance imaging. All of the patients will admitted to hospital to undergo physical examination, necessary lab examination, life quality questionnaire, cardiac magnetic resonance, 24 hours holter as follow-up at 3, 6, and 12 months.

6 Study Enrollment and Withdrawal

Target Sample Size	50 participants
Gender	Both genders
Age	35-80 years old

6.1 Subject Inclusion Criteria

In order to be eligible to participate in this study, an individual must meet all of the following criteria:

(1) Male or female, 35-80 years old.

(2) Coronary artery disease detected by coronary angiography not eligible for percutaneous coronary intervention.

(3) MRI confirmation of coronary artery disease and ischemic regions.

(4) LVEF ≤45% (assessed by 3D echocardiography).

(5) NYHA Class II-IV.

(6) No organ dysfunction in the lung, liver or kidney.

(7) Patients are able and willing to observe the therapeutic effects and adverse events.

(8) Signed informed consent was obtained.

(9) Negative serum pregnancy test.

(10) No coagulation dysfunction.

(11) Glycated hemoglobin ≤ 6.5.

6.2 Subject Exclusion Criteria

Any individual who meets any of the following criteria will be excluded from participation

in this study:

(1) Patients with ACS, including non-ST-segment elevation myocardial infarction (NSTEMI) and ST-segment elevation myocardial infarction (STEMI).

(2) Lactating or pregnant woman.

(3) Ineligibility for CABG.

(4) Unexplainable baseline laboratory abnormalities.

(5) Sensitivity to any of the study medications.

(6) Patients suffering from CVD, such as aortic disease, malignant arrhythmias, congenital heart disease, intra-cardiac mass, or moderate or severe valvular stenosis or regurgitation.

(7) History of life threatening allergic or immune-mediated reaction.

(8) Systemic infection or severe local infection.

(9) Shock, MODS or an inability to cooperate with doctors.

(10) Severe heart, lung, liver or renal dysfunction.

(11) Use of a medicine that might have an effect on the assessed outcomes.

(12) Suffering from HIV, Hepatitis B or Hepatitis C.

(13) Participation in any clinical trial in the past three months.

(14) History of mental illness or suicide risk.

(15) High expectation or unrealistic demands.

(16) Recent exposure to high levels of radiation.

(17) A previous or current history of neoplasia or other comorbidities that could impact the patient's short-term survival.

(18) Patients with serious complications of coronary artery disease (e.g., perforation of the interventricular septum, ventricular aneurysm or mitral regurgitation due to papillary muscle dysfunction).

(19) Abnormal coagulation function.

(20) Patients with hemodynamic instability that may lead to serious complications.

(21) Any condition that, in the judgment of the investigator, will place the patient at risk.

6.3 Strategies for Recruitment and Retention

Potential recruits may be identified through resident doctors and nurses in the Department of Thoracic and Cardiovascular Surgery, the Affiliated Drum Tower Hospital of Nanjing University Medical School, and referred to recruiting physicians (Dr. Qiang Wang and Dr. Xiaojun He) for evaluation. Patients will be screened to determine eligibility.

6.3.1Recruitment/informed consent

All eligible patients will be informed and talked about the clinical trial by recruiting physicians. After signed a statement of informed consent of the study, eligible patients were enrolled to the study and assigned randomly to collagen/cell group, cell group, and control group.

6.3.2Compensation

Subjects will be compensated for the time and inconvenience of participating in the study, including bill for operation and each follow-up (at 3, 6, 12months), which covered all the hospital admitted bill and round trip travel expense.

All the compensation was exempted from the hospital bills, which was completed by finance office.

6.4 Treatment Assignment Procedures

Enrolled participants will be assigned to treatment with approximately equal numbers of subjects randomized to collagen/cell group, cell group and control group. Subjects will be randomized ONLY after eligibility has been confirmed and informed consent has been obtained.

6.4.1 Randomization Procedures

Randomization was done as follow:

3 groups of random numbers were produced by excel software and encapsulated. Eligible patient drew the encapsulated random numbers and was assigned to different study group according to the random number.

6.4.2 Masking Procedures

Once cells were mixed with collagen scaffold, it formed white viscous solid-like hydrogel and need more force to inject into cardiac muscle than cells suspending in saline. Thus, the surgeon can distinguish cells with cell/collagen mixture during the injection process. So, it was difficult to blind the surgeons.

However, all of the assessments of safety and efficacy, including NYHA class assessment, MLHFQ questionnaire, 24 hours holter, lab and radiology examination were done by resident doctors and technicians blind to the study.

What's more, all of the participants were blinded to the study group.

6.5 Subject Withdrawal

Subjects may withdraw from the study voluntarily or be terminated by the principal investigator.

6.5.1 Reason for Withdrawal

Subjects are free to withdraw from participation in the study at any time upon request.

An investigator may terminate a subject's participation in the study if:

(1) Any serious adverse event (SAE) or other medical condition or situation occurs such that the subject cannot continue or continued participation in the study will not be in the best interest of the subject.

(2) The subject is deceased.

(3) The subject does not complete any follow-up.

(4) The study ends earlier than anticipated.

6.5.2Handing of Subject Withdrawals or Subject Discontinuation of Study Intervention

Subjects who are withdrawn from the study, either through voluntary discontinuation, for failure to complete study follow-up, or by decision of the clinical site principal investigator for reasons enumerated above, will be recorded as terminated. They will no longer be followed or be brought back for a follow-up hospitalization.

If a subject is withdrawn due to an SAE or other medical condition that precludes their continuation in the study, the site PI will call the subject to discuss and ensure appropriate medical treatment or follow up is arranged.

The study sample size has been calculated to allow for attrition due to early withdrawal or discontinuation.

6.6 Premature Termination or Suspension of Study

The study may be suspended or prematurely terminated if there is sufficient reason or cause. Written notification, documenting the reason for study suspension or termination, will be provided by the suspending or terminating party to the investigator, funding agency, and regulatory authorities. If the study is prematurely terminated or suspended, the site principal investigator will promptly inform the IRB and will provide the reason(s) for the termination or suspension.

7 Baseline assessment

Patients' baseline characteristics were collected after informed consent document was signed by participants themselves.

The baseline characteristics include:

（1） Demographics and medical history (Male sex, Pre-operation hospital days, Total hospital days, Age, years, Hypertension, Hyperlipidaemia, Diabetes mellitus, Tobacco use, Alcohol use, Previous PCI, Stroke, Peripheral artery disease, Hepatic disease);

（2） Physical examination (Body temperature, Heart rate, Breath, Systolic blood pressure, Diastolic blood pressure, SpO2(%), Body Weight, Body height, Body-mass index);

（3） Duration from symptom to operation (≤ 30 days, No. (%))

（4） Infarct artery distribution (left main artery, left anterior descending artery, left circumflex artery, right coronary artery);

(5) Number of vessels >50% (None, One-vessel, Two-vessel, Three-vessel, More than three-vessel);

(6) Prior infarction area (Apex infarction, Anterior infarction, Free wall infarction, Posterior infarction, Inferior infarction, Septal infarction);

(7) Medication before initial hospitalization (aspirin, clopidogrel, statin, β blocker, ACEI or ARB, nitrate, calcium channel blockers);

(8) Complication (hydrothorax, Pulmonary infection, hydropericardium, seroperitoneum);

(9) Cardia function (LVEF (3D echo) below average, LVEF(CMR) below average);

(10) NYHA Class;

(11) Myocardial damage marker (CK-MB, cTnT).

(12) MLHFQ score;

(13) 24 hours holter;

(14) Laboratory examination (immunoglobulin, renal function, liver function, inflammation markers).

8 Study Intervention

8.1 Study Procedural Interventions Description

Patients will be randomized to either collagen/cell group, cell group or control group. Operative and medical interventions performed for this study are consistent with routine clinical standards of CABG treatment. The cardiac infarcted area was confirmed with echocardiography, MRI and coronary angiography.

In collagen/cell group, hUC-MSCs (1×10^8/1.5 ml PBS) was mixed with 1 ml of the injectable collagen hydrogel. The mixture was then injected into cardiac muscle under direct vision after completion of CABG and before close chest. 5-10 points in the central and border areas of all infarcted region were chosen as injection sites.

In cell group, hUC-MSCs (1×10^8/2.5 ml PBS) was injected as the same method in collagen/cell group.

8.2 Procedures for Training of Clinicians on Procedural Intervention

8.2.1 Treating Surgeons

All study surgeons will follow the standardized surgical protocol.

8.2.2 Treating clinical doctors and nurses

All resident doctors and nurses in the trial site (Department of Thoracic and Cardiovascular Surgery, the Affiliated Drum Tower Hospital of Nanjing University Medical School) -- whom were unacquainted with the study – were trained by the recruiting physician to aware of potential eligible participants. They were briefed with including criteria of the study and reported to recruiting physician once they found potential target patients.

9 Follow-Up

Subjects enrolled in the study and randomized to treatment will be asked to re-admitted to the trial clinical site to complete follow-up assessment at 3, 6, 12 months after treatment. The allowed window for completion of follow-up hospitalization is -3 weeks/+3 weeks from the ideal follow-up time point.

Assessment during follow-up hospitalization are:

（1） Demographics and medical history

（2） Physical examination

（3） NYHA Class;

（4） Myocardial damage marker (CK-MB, cTnT).

（5） MLHFQ score;

（6） 24 hours holter;

（7） Laboratory examination (immunoglobulin, renal function, liver function, inflammation markers).

（8） Cardiac magnetic resonance imaging.

10 Study closure

10.1 End of Study

For regulatory purposes the end of the trial will be 12 months after randomization of the last patient or the death of the last surviving patient, whichever event occurs first. At this point the ‘declaration of end of trial’ form will be submitted to the ethics committee, as required.

Following this, IRB will advise sites on the procedure for closing the trial at the

site.

10.2 Early discontinuation of study

The trial may be stopped before completion as an Urgent Safety Measure on the recommendation of the IRB. Sites will be informed in writing by IRB of reasons for early closure and the actions to be taken with regard to the treatment and follow up of patients.

11 Assessment of Safety

11.1 Specification of Safety Parameters

11.1.1 Unanticipated Problems

Unanticipated problems include breach of confidentiality or loss of data. Safeguards to prevent this include storage of data in locked file cabinets, use of password protected databases, multi backup of data files, and restricting access to data by authorized study personnel only.

11.1.2 Adverse Events (AEs)

AEs for this trial include:

- Post-operative infection
- Post-operative bleeding
- Post-operative inflammation
- Complications due to anesthesia

11.1.3 Serious Adverse Events (SAEs)

SAEs for this trial include:

- all-cause death,
- post-operation MI,
- new tumors,
- systemic infection,
- stroke,
- hospitalization because of HF,
- cardiac perforation,
- pericardial tamponade,
- ischemia,
- anaphylaxis,
- hemodynamic instability,
- sustained ventricular arrhythmias (>30 s or causing hemodynamic compromise).

11.2 Time Period and Frequency for Event ASSESSMENT AND Follow-Up

All reportable events will be recorded in the study record with start dates occurring any time after informed consent is obtained until 7 days (for non-serious AEs) or 30 days (for SAEs) after the last day of study participation for the study (12 months of follow-up). Subjects will be asked about the occurrence of AE/SAEs during each follow-up hospitalization.

Events will be followed for outcome information until resolution or stabilization, as appropriate. Unanticipated problems will be recorded in the data collection system throughout the study as discovered.

11.3 Characteristics of an Adverse Event

11.3.1 Relationship to Study Intervention

Study PI will assess relationship to study intervention. To assess relationship of an event to study intervention, the following guidelines will be used:

1. Related (Possible, Probable, Definite)

a. The event is known to occur with the study intervention.

b. There is a temporal relationship between the intervention and event onset.

c. The event abates when the intervention is discontinued.

d. The event reappears upon a re-challenge with the intervention.

2. Not Related (Unlikely, Not Related)

a. There is no temporal relationship between the intervention and event onset.

b. An alternate etiology has been established.

11.3.2 Expectedness of Event

The Study PI will be responsible for determining whether an adverse event is expected or unexpected. An adverse event will be considered unexpected if the nature, severity, or frequency of the event is not consistent with the risk information for the intervention.

11.3.3 Severity of Event

The study PI will determine severity of event. The following scale will be used to grade adverse events:

1. Mild: no intervention required; no impact on activities of daily living (ADL)

2. Moderate: minimal, local, or non-invasive intervention indicated; moderate impact on ADL

3. Severe: significant symptoms requiring invasive intervention; subject seeks medical attention, needs major assistance with ADL.

11.4Reporting Procedures

11.4.1 Related SAEs & Safety Issues

The following will be reported to the IRB within 7 calendar days of the PI becoming aware of the event:

- Any serious adverse event that in the investigator’s opinion was unanticipated or unexpected, involved risk to participants or others, and was possibly related to the research procedures
- Any noncompliance with the IRB-approved protocol that increased risk or affected the participant’s rights, safety, or welfare.

11.4.2 AEs & Unrelated SAEs

Anticipated AEs or unrelated SAEs will be handled in a less urgent manner but will be reported to the IRB.

All individual AE and Safety reports will be maintained by the investigator and a summary (not individual reports) of all adverse events that have occurred within the last approval period that are associated with the study, whether related or non-related, will be submitted to the IRB at the time of continuing review.

Adverse events may include a participant's death as a result of a longtime illness (non-related), a breach in confidentiality, or any complaint of a participant unless the risk involved is serious (in which case, the event is reported as an unanticipated problem involving risk to participants or others or as a serious adverse event at the time of occurrence).

11.4.3 Unanticipated Problems

Incidents or events for unanticipated problems will be recorded in an event report form. The following information will be included when reporting an adverse event, or any other incident, experience, or outcome as an unanticipated problem to the IRB:

- appropriate identifying information for the research protocol, such as the title, investigator's name, and the IRB project number;
- a detailed description of the adverse event, incident, experience, or outcome;
- an explanation of the basis for determining that the adverse event, incident, experience, or outcome represents an unanticipated problem;
- a description of any changes to the protocol or other corrective actions that have been taken or are proposed in response to the unanticipated problem.

To satisfy the requirement for prompt reporting, unanticipated problems will be reported using the following timeline:

- Unanticipated problems that are serious adverse events will be reported to the IRB within 7 calendar days of the investigator becoming aware of the event.
- Any other unanticipated problem will be reported to the IRB, in a less urgent manner during annual continuing review.

11.5Halting Rules

12 Study Oversight

In addition to the PI's responsibility for oversight, study oversight will be under the direction of the IRB. The IRB will meet annually, or more frequently at their discretion, to assess safety

and efficacy data, study progress, and data integrity for the study. If safety concerns arise, more frequent meetings may be held.

Otherwise, IRB will monitor trial operations, ensure adequate study accrual, protocol compliance, timely data entry, ensure implementation of the recommendations of the IRB, and general study integrity. At the end of each meeting the IRB will make a formal recommendation regarding the continuation of the study.

12.1Study management Group (SMG)

Study management group include the recruiting physicians (Dr. Xiaojun He, Dr. He Zhang, and Dr. Qiang Wang). The management group was in charge of evaluation, recruiting, follow-up of participants, and coordinate of the Department of Thoracic and Cardiovascular Surgery, Center for Clinical Stem Cell Research, Department of Cardiology, and Department of Radiology.

13 Clinical Site Monitoring

Internal clinical site monitoring will be conducted to ensure that the rights of human subjects are protected, that the study is implemented in accordance with the protocol and/or other operating procedures, and that the quality and integrity of study data and data collection methods are maintained. Monitoring for this study will be performed by the study PI and by the IRB, who will review data and documentation regularly and visit sites as needed.

13.1Screening/Enrollment Monitoring Reports

The screening and enrollment data for clinical site will be reviewed monthly by the clinical site PI and study PI to ensure that potentially eligible study participants are being screened, approached and consented as appropriate and study-related documents and images are submitted in accordance with the study protocol.

13.2Site Monitoring Calls

Clinical sites will participate in regular site calls with the executive study coordinator to review data from Screening, Enrollment & Data reports. Call participants, notes regarding what was discussed, and any actions to be taken will be recorded and reviewed periodically by the study PI. Issues of immediate concern will be brought to the attention of the study PI as they are identified.

13.3Site Visits

If issues or questions of concern pertaining to site operations arise during the course of the study, the study PI &/or executive study coordinator may visit the site in person to observe

how site procedures are being implemented and determine appropriate remedies, if needed.

Sites may also be visited within the first year of recruitment to ensure everything is operating correctly and to address any operational questions or challenges, as deemed appropriate by the study PI.

14 Statistical Considerations

14.1Study Hypotheses

Aim 1: To explore the safety of collagen hydrogel mixed mesenchymal stromal cells transplantation in patients undergoing coronary artery bypass operation during 12 months' follow-up.

Hypothesis 1: the cell laden collagen gel intramyocardial injection was safe in CHID patients under CABG operation.

Aim 2: To assess effects of collagen hydrogel mixed mesenchymal stromal cells transplantation in patients undergoing coronary artery bypass operation during 12 months' follow-up.

Hypothesis 2: CHID patients treated with CABG plus cell laden collagen gel injection will have higher LVEF and smaller scar size at 12 months after operation.

14.2Sample Size Considerations

Power calculation of sample size was performed according to the previous studies[30-33]. Previous studies assumed an average infarct size of total LV mass is between 15%-25%, and the expected treatment effect reduction of is 4-10 absolute percentage points. And the standard deviation (SD) of myocardial infarct size is 8%-10%. So with a risk of type 1 error of 5% and type 2 error of 20%, we wanted to find 9 absolute percentage point reduction in infarct size. Assuming an SD of 8, we needed 14 patients each group. Considering the possible dropout, the total sample size was determined to be 50 patients for 3 groups.

15 Source Documents & Access to Source Data/Documents

Study staff will maintain appropriate medical and research records for this study. Study staff will permit authorized examination (and when required by applicable law, to copy) of research records for the purposes of quality assurance reviews, audits, and evaluation of the study safety, progress, and data validity.

15.1Source Documents

This study will utilize the following source documents:

- Patient medical records, including clinic notes, surgery notes, and nursing records;
- Reports of MRI, echocardiography, 24 hours holter, and lab examination.
- Informed consent documents
- Payment records of compensation for participants.

15.2 Maintenance of and Access to Source Documents

15.2.1 Consent Forms

A copy of the signed consent form will be provided to the subject.

15.2.2 Case Report Forms

A case report form will be initiated for potentially eligible patients whose available medical records do not indicate the presence of exclusionary factors. Forms will include basic demographic information.

For subjects determined to be ineligible or who decline to participate, information from the case report form will be entered into the screening database, including the reason for ineligibility &/or non-enrollment. These documents will not be retained.

The original hard copy will be kept on file in a locked cabinet where it was originally completed/collected (accessible only to authorized local site study personnel)

16 Quality Control and Quality Assurance

16.1 Electronic Data Capture

Patients information include baseline characteristics, operation parameters, follow-up data will be collected and organized as excel files (only to authorized local site study personnel).

16.2 Data Entry

Dr. He Zhang was in charge of data entry and Dr. Xiaojun He was responsible for data validation and statistical analysis.

17 Ethics/Protection of Human Subjects

17.1 Ethical Standard

The study was performed in accordance with the principles of the Declaration of Helsinki.

17.2 Ethical approval

The protocol, informed consent form(s), recruitment materials, and all subject materials will be submitted to the IRB for review and approval. Approval of both the protocol and the consent form must be obtained before any subject is enrolled. Any amendment to the protocol will require review and approval by the IRB before the changes are implemented in the study.

Review and approval of the protocol and supporting materials for this study will be conducted by the institutional review board of the Ethics Committee of Nanjing University Medical School Affiliated Nanjing Drum Tower Hospital in China.

The IRBs will ensure compliance with local laws, institutional policies, and govern regulations.

17.3 Informed Consent Process

Informed consent is a process that is initiated prior to the individual agreeing to participate in the study and continues throughout study participation. Extensive discussion of risks and possible benefits of study participation will be provided to subjects and their families, if applicable. A consent form describing in detail the study procedures and risks will be given to the subject. Consent forms will be IRB-approved, and the subject is required to read and review the document or have the document read to him or her. The investigator or designee will explain the research study to the subject and answer any questions that may arise. The subject will sign the informed consent document prior to any study-related assessments or procedures. Subjects will be given the opportunity to discuss the study with their surrogates or think about it prior to agreeing to participate. They may withdraw consent at any time throughout the course of the study. A copy of the signed informed consent document will be given to subjects for their records. The rights and welfare of the subjects will be protected by emphasizing to them that the quality of their clinical care will not be adversely affected if they decline to participate in this study. The consent process will be documented in the research record.

17.4 Subject Confidentiality and data protection

Subject confidentiality is strictly held in trust by the investigators, and hospital staff. This confidentiality is extended to cover medical imaging and tests in addition to any study information relating to subjects.

The study protocol, documentation, data, and all other information generated will be held in strict confidence. No information concerning the research study or the data will be released to any unauthorized third party.

The study monitor, auditor(s), and authorized representatives of the IRB, may inspect all study documents and records required to be maintained by the investigators, including, but not limited to, medical records (office, clinic, or hospital) for the study subjects. The clinical study sites will permit access to such records.

18 Literature References

1. Yusuf S, Zucker D, Peduzzi P, Fisher LD, Takaro T, Kennedy JW, Davis K, Killip T, Passamani E, Norris R and et al. Effect of coronary artery bypass graft surgery on survival: overview of 10-year results from randomised trials by the Coronary Artery Bypass Graft Surgery Trialists Collaboration. *Lancet*. 1994;344:563-70.

2. Jeremias A, Kaul S, Rosengart TK, Gruberg L and Brown DL. The impact of revascularization on mortality in patients with nonacute coronary artery disease. *The American journal of medicine*. 2009;122:152-61.

3. Windecker S, Stortecky S, Stefanini GG, da Costa BR, Rutjes AW, Di Nisio M, Silletta MG, Maione A, Alfonso F, Clemmensen PM, Collet JP, Cremer J, Falk V, Filippatos G, Hamm C, Head S, Kappetein AP, Kastrati A, Knuuti J, Landmesser U, Laufer G, Neumann FJ, Richter D, Schauerte P, Sousa Uva M, Taggart DP, Torracca L, Valgimigli M, Wijns W, Witkowski A, Kolh P and Juni P. Revascularisation versus medical treatment in patients with stable coronary artery disease: network meta-analysis. *BMJ (Clinical research ed)*. 2014;348:g3859.

4. Senyo SE, Steinhauser ML, Pizzimenti CL, Yang VK, Cai L, Wang M, Wu TD, Guerquin-Kern JL, Lechene CP and Lee RT. Mammalian heart renewal by pre-existing cardiomyocytes. *Nature*. 2013;493:433-6.

5. Jeevanantham V, Butler M, Saad A, Abdel-Latif A, Zuba-Surma EK and Dawn B. Adult Bone Marrow Cell Therapy Improves Survival and Induces Long-Term Improvement in Cardiac Parameters A Systematic Review and Meta-Analysis. *Circulation*. 2012;126:551-+.

6. Orlic D, Kajstura J, Chimenti S, Jakoniuk I, Anderson SM, Li B, Pickel J, McKay R, Nadal-Ginard B, Bodine DM, Leri A and Anversa P. Bone marrow cells regenerate infarcted myocardium. *Nature*. 2001;410:701-5.

7. Chong JJH, Yang XL, Don CW, Minami E, Liu YW, Weyers JJ, Mahoney WM, Van Biber B, Cook SM, Palpant NJ, Gantz JA, Fugate JA, Muskheli V, Gough GM, Vogel KW, Astley CA, Hotchkiss CE, Baldessari A, Pabon L, Reinecke H, Gill EA, Nelson V, Kiem HP, Laflamme MA and Murry CE. Human embryonic-stem-cell-derived cardiomyocytes regenerate non-humanprimate hearts. *Nature*. 2014;510:273-+.

8. Liang XT, Ding Y, Zhang YL, Tse HF and Lian QZ. Paracrine Mechanisms of Mesenchymal Stem Cell-Based Therapy: Current Status and Perspectives. *Cell Transplant*. 2014;23:1045-1059.

9. Menasche P, Alfieri O, Janssens S, McKenna W, Reichenspurner H, Trinquart L, Vilquin JT, Marolleau JP, Seymour B, Larghero J, Lake S, Chatellier G, Solomon S, Desnos M and Hagege AA. The Myoblast Autologous Grafting in Ischemic Cardiomyopathy (MAGIC) trial: first randomized placebo-controlled study of myoblast transplantation. *Circulation*. 2008;117:1189-200.

10. Menasche P, Hagege AA, Scorsin M, Pouzet B, Desnos M, Duboc D, Schwartz K, Vilquin JT and Marolleau JP. Myoblast transplantation for heart failure. *Lancet*. 2001;357:279-80.

11. Ang KL, Chin D, Leyva F, Foley P, Kubal C, Chalil S, Srinivasan L, Bernhardt L, Stevens S, Shenje LT and Galinanes M. Randomized, controlled trial of intramuscular or intracoronary injection of autologous bone marrow cells into scarred myocardium during CABG versus CABG alone. *Nat Clin Pract Cardiovasc Med*. 2008;5:663-70.

12. Hendrikx M, Hensen K, Clijsters C, Jongen H, Koninckx R, Bijnens E, Ingels M, Jacobs A, Geukens R, Dendale P, Vijgen J, Dilling D, Steels P, Mees U and Rummens JL. Recovery of regional but not global contractile function by the direct intramyocardial autologous bone marrow transplantation: results from a randomized controlled clinical trial. *Circulation*. 2006;114:I101-7.

13. Nasseri BA, Ebell W, Dandel M, Kukucka M, Gebker R, Doltra A, Knosalla C, Choi YH, Hetzer R and Stamm C. Autologous CD133+ bone marrow cells and bypass grafting for regeneration of ischaemic myocardium: the Cardio133 trial. *Eur Heart J*. 2014;35:1263-74.

14. Stamm C, Kleine HD, Choi YH, Dunkelmann S, Lauffs JA, Lorenzen B, David A, Liebold A, Nienaber C, Zurakowski D, Freund M and Steinhoff G. Intramyocardial delivery of CD133+ bone marrow cells and coronary artery bypass grafting for chronic ischemic heart disease: safety and efficacy studies. *J Thorac Cardiovasc Surg*. 2007;133:717-25.

15. Malliaras K, Makkar RR, Smith RR, Cheng K, Wu E, Bonow RO, Marban L, Mendizabal A, Cingolani E, Johnston PV, Gerstenblith G, Schuleri KH, Lardo AC and Marban E. Intracoronary cardiosphere-derived cells after myocardial infarction: evidence of therapeutic regeneration in the final 1-year results of the CADUCEUS trial (CArdiosphere-Derived aUtologous stem CElls to reverse ventricUlar dySfunction). *J Am Coll Cardiol*. 2014;63:110-22.

16. Smith RR, Barile L, Cho HC, Leppo MK, Hare JM, Messina E, Giacomello A, Abraham MR and Marban E. Regenerative potential of cardiosphere-derived cells expanded from percutaneous endomyocardial biopsy specimens. *Circulation*. 2007;115:896-908.

17. van Berlo JH, Kanisicak O, Maillet M, Vagnozzi RJ, Karch J, Lin SC, Middleton RC, Marban E and Molkentin JD. c-kit+ cells minimally contribute cardiomyocytes to the heart. *Nature*. 2014;509:337-41.

18. Ascheim DD, Gelijns AC, Goldstein D, Moye LA, Smedira N, Lee S, Klodell CT, Szady A, Parides MK, Jeffries NO, Skerrett D, Taylor DA, Rame JE, Milano C, Rogers JG, Lynch J, Dewey T, Eichhorn E, Sun B, Feldman D, Simari R, O'Gara PT, Taddei-Peters WC, Miller MA, Naka Y, Bagiella E, Rose EA and Woo YJ. Mesenchymal precursor cells as adjunctive therapy in recipients of contemporary left ventricular assist devices. *Circulation*. 2014;129:2287-96.

19. Hou D, Youssef EA, Brinton TJ, Zhang P, Rogers P, Price ET, Yeung AC, Johnstone BH, Yock PG and March KL. Radiolabeled cell distribution after intramyocardial, intracoronary, and interstitial

retrograde coronary venous delivery: implications for current clinical trials. *Circulation*. 2005;112:I150-6.

20. Christman KL, Vardanian AJ, Fang Q, Sievers RE, Fok HH and Lee RJ. Injectable fibrin scaffold improves cell transplant survival, reduces infarct expansion, and induces neovasculature formation in ischemic myocardium. *J Am Coll Cardiol*. 2004;44:654-60.

21. Dai W, Hale SL, Kay GL, Jyrala AJ and Kloner RA. Delivering stem cells to the heart in a collagen matrix reduces relocation of cells to other organs as assessed by nanoparticle technology. *Regen Med*. 2009;4:387-95.

22. Kutschka I, Chen IY, Kofidis T, von Degenfeld G, Sheikh AY, Hendry SL, Hoyt G, Pearl J, Blau HM, Gambhir SS and Robbins RC. In vivo optical bioluminescence imaging of collagen-supported cardiac cell grafts. *J Heart Lung Transplant*. 2007;26:273-80.

23. Liu Z, Wang H, Wang Y, Lin Q, Yao A, Cao F, Li D, Zhou J, Duan C, Du Z, Wang Y and Wang C. The influence of chitosan hydrogel on stem cell engraftment, survival and homing in the ischemic myocardial microenvironment. *Biomaterials*. 2012;33:3093-106.

24. Martens TP, Godier AF, Parks JJ, Wan LQ, Koeckert MS, Eng GM, Hudson BI, Sherman W and Vunjak-Novakovic G. Percutaneous cell delivery into the heart using hydrogels polymerizing in situ. *Cell Transplant*. 2009;18:297-304.

25. Wang H, Shi J, Wang Y, Yin Y, Wang L, Liu J, Liu Z, Duan C, Zhu P and Wang C. Promotion of cardiac differentiation of brown adipose derived stem cells by chitosan hydrogel for repair after myocardial infarction. *Biomaterials*. 2014;35:3986-98.

26. Wang T, Jiang XJ, Tang QZ, Li XY, Lin T, Wu DQ, Zhang XZ and Okello E. Bone marrow stem cells implantation with alpha-cyclodextrin/MPEG-PCL-MPEG hydrogel improves cardiac function after myocardial infarction. *Acta Biomater*. 2009;5:2939-44.

27. Yu J, Du KT, Fang Q, Gu Y, Mihardja SS, Sievers RE, Wu JC and Lee RJ. The use of human mesenchymal stem cells encapsulated in RGD modified alginate microspheres in the repair of myocardial infarction in the rat. *Biomaterials*. 2010;31:7012-20.

28. Gelse K, Poschl E and Aigner T. Collagens--structure, function, and biosynthesis. *Adv Drug Deliv Rev*. 2003;55:1531-46.

29. Perea-Gil I, Prat-Vidal C and Bayes-Genis A. In vivo experience with natural scaffolds for myocardial infarction: the times they are a-changin'. *Stem Cell Res Ther*. 2015;6:248.

30. Freixa X, Bellera N, Ortiz-Pérez JT, Jiménez M, Paré C, Bosch X, De Caralt TM, Betriu A and Masotti M. Ischaemic postconditioning revisited: lack of effects on infarct size following primary percutaneous coronary intervention. *Eur Heart J*. 2012;33:103-12.

31. Lønborg J, Kelbaek H, Vejlstrup N, Jørgensen E, Helqvist S, Saunamäki K, Clemmensen P, Holmvang L, Treiman M, Jensen JS and Engstrøm T. Cardioprotective effects of ischemic postconditioning in patients treated with primary percutaneous coronary intervention, evaluated by magnetic resonance. *Circulation Cardiovascular interventions*. 2010;3:34-41.

32. Siddiqi N, Neil C, Bruce M, MacLennan G, Cotton S, Papadopoulou S, Feelisch M, Bunce N,

Lim PO, Hildick-Smith D, Horowitz J, Madhani M, Boon N, Dawson D, Kaski JC and Frenneaux M. Intravenous sodium nitrite in acute ST-elevation myocardial infarction: a randomized controlled trial (NIAMI). *Eur Heart J*. 2014;35:1255-62.

33. Song YB, Hahn JY, Gwon HC, Kim JH, Lee SY, Choe YH, Choi SH, Choi JH and Lee SH. Upstream high-dose tirofiban does not reduce myocardial infarct size in patients undergoing primary percutaneous coronary intervention: a magnetic resonance imaging pilot study. *Clin Cardiol*. 2009;32:321-6.
