# Supplementary material for: Effect of Intramyocardial Grafting Collagen Scaffold With Mesenchymal Stromal Cells in Patients With Chronic Ischemic Heart Disease: A Randomized Clinical Trial
Source: JAMA Netw Open. 2020 Sep 10;3(9):e2016236. doi: 10.1001/jamanetworkopen.2020.16236 (PMC7489863; doi:10.1001/jamanetworkopen.2020.16236)
Supplement: Supplement 2. — eMethods. Materials and Methods eFigure 1. Characterization of hUC-MSCs eFigure 2. Laboratory Results of Safety Assessment eFigure 3. Restoration of Cardiac Function and Attenuation of Negative LV Remodeling eFigure 4. Blood Cell Count and Body Weight Change eFigure 5. Representative Cardiac Magnetic Resonance Images eFigure 6. Analysis of Immune Cells in Mouse Spleen After Collagen Hydrogel Analysis eTable 1. CMR and MLHFQ Data (Safety Analysis Set) eTable 2. Parameters of CABG Operation eTable 3. Intradermal Irritation Assay eTable 4. Inclusion Criteria eTable 5. Exclusion Criteria [file jamanetwopen-e2016236-s002.pdf]

Supplementary Online Content

He X, Wang Q, Zhao Y, et al. Effect of intramyocardial grafting collagen scaffold with mesenchymal stromal cells in patients with chronic ischemic heart disease: a randomized clinical trial. *JAMA Netw Open*. 2020;3(9):e2016236. doi:10.1001/jamanetworkopen.2020.16236

eMethods. Materials and Methods

eFigure 1. Characterization of hUC-MSCs

eFigure 2. Laboratory Results of Safety Assessment

eFigure 3. Restoration of Cardiac Function and Attenuation of Negative LV Remodeling

eFigure 4. Blood Cell Count and Body Weight Change

eFigure 5. Representative Cardiac Magnetic Resonance Images

eFigure 6. Analysis of Immune Cells in Mouse Spleen After Collagen Hydrogel Analysis

eTable 1. CMR and MLHFQ Data (Safety Analysis Set)

eTable 2. Parameters of CABG Operation

eTable 3. Intradermal Irritation Assay

eTable 4. Inclusion Criteria

eTable 5. Exclusion Criteria

This supplementary material has been provided by the authors to give readers additional information about their work.

© 2020 He X et al. *JAMA Network Open.*

eMethods. Materials and Methods

End Points

The primary endpoint was the safety of the homologous, allogeneic hUC-MSCs and collagen scaffolds, as assessed by the incidence of serious adverse events (SAE) [time frame of up to 12 months after surgery], which were defined as a composite of all-cause death, post-operation MI, new tumors, sustained ventricular tachycardia, systemic infection, stroke, allergic reaction, hospitalization because of HF, cardiac perforation, pericardial tamponade, ischemia, anaphylaxis, hemodynamic instability, or sustained ventricular arrhythmias (>30 s or causing hemodynamic compromise). Additional safety assessments included the clinical monitoring of adverse events (AE), changes in vital signs, electrocardiogram (ECG) and laboratory values (CRP [C-reactive protein], CK-MB, hematology, chemistry, and urinalysis).

The secondary endpoint was the efficacy of hUC-MSCs and collagen scaffold, as assessed according to the CMR-based LVEF and infarct size at 3, 6 and 12 months after treatment. The additional exploratory secondary efficacy end points were based on CMR (indices of ventricular remodeling and function) and clinical assessments (the New York Heart Association (NYHA) class and the MLHFQ) at 3, 6 and 12 months after treatment. NYHA class was assessed by clinical doctors who are blinded to the whole study. MLHFQ was completed by patients themselves who are blinded.

Patients

The study protocol was approved by the institutional review board of the Ethics Committee of Nanjing University Medical School Affiliated Nanjing Drum Tower Hospital, Nanjing, Jiangsu, China. All patients agreed to participate and signed a statement of informed consent approved by the institutional review board before enrollment. The study was performed in accordance with the principles of the Declaration of Helsinki.

The protocol is provided in Figure 1. Target population was CIHD patients with left ventricular ejection fraction (LVEF) ≤ 45% (assessed by 3D echocardiography) and who needed CABG and not suitable for percutaneous coronary

© 2020 He X et al. *JAMA Network Open.*

intervention revascularization, which means CIHD complex with LM stenosis >50%, proximal LAD stenosis >50%, or two or three-vessel stenosis >50% with impaired LV function[33]. Enrollment was based on eligibility screening at the beginning of the hospital period. Other inclusion and exclusion criteria are provided in Supplementary Table 5 and 6.

Cell Preparation

Neonatal human umbilical cords were obtained from the Gynecology and Obstetrics Department and delivered to the Good Manufacturing Practice (GMP) facility of Clinical Stem Cell Research Center of Nanjing University Medical School Affiliated Nanjing Drum Tower Hospital. The hUC-MSCs were derived from a single eligible donor, isolated from Wharton's jelly in neonate human umbilical cords and expanded by methods described previously. Cells were seeded in Dulbecco's modified Eagle medium (Gibco) with 10% fetal bovine serum (FBS) and cultured in 5% CO2 at 37°C. 3 days later, the medium was replaced to remove the non-adherent cells. Cells were passaged when they reached confluence. Surface markers of hUC-MSCs were assessed by a cytomics FC500 flow cytometer (BD Biosciences). Differentiation potential of bone, adipocytes and cartilage was routinely assessed by Alizarin Red S staining, Oil Red O staining and Alcian Blue staining.

Stringent quality assessment of the hUC-MSCs was taken based on our established quality evaluation system for MSCs, including the donor screening criteria, cell safety, quality, and biological effects, not only in line with the basic criteria of biological products, but also following the general requirements of drugs. The hUC-MSCs cells were judged as qualified cell-therapeutic products for clinic use when they met all the requirements and standards of clinic-graded MSCs.

The same batch MSCs at third passage were cryo-preserved into many frozen tubes in liquid nitrogen. The cryo-preserved MSCs passing the strict quality assessment were named as Working Bank Cells. Prior to a patient needed the MSC transplant, according to the amount of MSC transplant, several frozen tubes of MSCs were thawed and passage one time. Cells reached confluence and then they were undergone a release examination. Thus MSCs at fifth passage could be used for patient only they pass both the systematic quality assessments and release examination. Then the cells were transported to the operation room for injection during surgery.

© 2020 He X et al. *JAMA Network Open.*

Collagen Gel Preparation

The injectable collagen scaffolds were obtained from bovine tissues. Fresh bovine aponeuroses were harvested and treated with 1% tri(n-butyl) phosphate for 48 h. Cells and other proteins were removed by immersed in 1% trypsin for 1 hour. The collagen was then washed with deionized water, and dissolved in 0.5 M acetic acid for 24 h and dialyzed in deionized water for 10 days, after which it was lyophilized and dissolved in saline to form a 30mg/ml injectable collagen scaffold.

Injectable collagen scaffolds were lyophilized, sputter-coated with gold and observed by scanning electron microscopy (SEM). The rheological characteristics of the collagen scaffold mixed with cells were quantified by monitoring the storage moduli (G′) and loss moduli (G″) using a DHR-2 rheometer (TA instrument) at 25 °C in dynamic oscillatory mode with a frequency sweep from 0.1 to 1 Hz at 1% strain (Figure 2).

Allergen detection and assays for viral contamination, acute toxicity, cytotoxicity, subchronic toxicity, intradermal irritation, genetic toxicity, hemolytic toxicity, and degradation were performed by the National Institute of Food and Drug Control according to the Chinese Criterion for Medical Devices GB16886 to evaluate the biological safety of the injectable collagen scaffold (Figure 2).

Collagen Biosafety Evaluation

For acute toxicity, collagen scaffolds were incubated in saline for 72 hours according to the ratio of 0.2g/ml. Then saline after incubation was injected into mice with the ratio of 50ml/kg. The weight of mice (eFigure 3), general observation of animal behavior and death were recorded during 72 hours. There is no death or abnormal behavior of mice was observed in our study.

For cytotoxicity assay, collagen scaffolds were incubated in DMEM cell culture medium with 10% fetal bovine serum for 24 hours according to the ratio of 0.2g/ml. Then the medium after incubation with scaffolds was added into L929 cells to detect the cell proliferation rates during 24 hours by MTT assay. The cell proliferation rate is over 80% in our study.

© 2020 He X et al. *JAMA Network Open.*

For intradermal irritation, collagen scaffolds were incubated in saline or oil for 72 hours according to the ratio of 0.2g/ml. 200ul saline or oil after incubation was injected into rabbit skin. The erythema and edema of rabbit skin was recorded during 72 hours. There is no significant difference was found in saline or oil groups compared to control groups suggesting the scaffold has no intradermal irritation (eTable 3).

The genetic toxicity of extracted liquid of scaffold was evaluated by Ames assay and TK gene mutant assay after collagen scaffolds were incubated in saline for 72 hours according to the ratio of 0.2g/ml. No genetic toxicity was found in our study.

For hemolytic toxicity, collagen scaffolds were incubated in saline for 72 hours according to the ratio of 0.2g/ml, then the saline was added into anticoagulant rabbit serum for 60min, and the absorbance at 545nm was recorded. The hemolysis rate of collagen scaffold in our study is about 0.6%.

For immune response assay, 13ul collagen hydrogel was subcutaneously injected into mice (20g), and 100ul BSA-Freund adjuvant was injected as positive control, and sham mice was used as negative control. After 28 days, blood cell counting (Figure 2E) was detected by Automated Hematology Analyzer and the serum IgG and IgM (Figure 2F) was determined by Elisa kit. Cells in spleen was isolated and detected by Flow cytometry (Figure 2G). Antibodies for CD3, CD4, CD8a, CD19, CD45, CD69 and CD49b were used to identify different immune cells.

Randomization and Masking

Randomization was performed by two investigators (Xiaojun He and He Zhang) before the final eligibility screening. The numbers assigned to patients were entered into a computer software program that randomly allocated the treatment assignment with a 1:1:1 ratio by use of an adaptive block randomization scheme (Figure 1). Xiaojun He and He Zhang assigned the patients. The investigators performing the echocardiographic and CMR analyses were masked to the group assignments.

CMR

CMR images were acquired using a 1.5T scanner (Philips Achieva, Cleveland, OH) with Syngo magnetic resonance software. Analyses of the LVEF, left ventricular end diastole volume (LVEDV), left ventricular end systolic volume (LVESV) and wall motion were performed with the Cardiac Explorer software package. Delayed contrast-enhanced imaging for infarct assessment was also performed. Infarct size was automatically calculated as the ratio of the area of myocardium with delayed enhancement to the area of LV myocardium in each slice from the short-axis delayed-enhancement images. Total infarct size was calculated by summation of all slice volumes with hyper-enhancement and divided by slice number, which was expressed in g. Assessment of the infarct size was also performed semi-quantitatively (with a standard transmural categorization score[17] of 1–4, with 1 representing no infarct, 2 representing less than 25% transmural involvement, 3 representing 25–50%, and 4 representing more than 50%).

Statistical Analysis

All values were expressed as the mean ± standard deviation unless otherwise stated. Equal distribution was tested with Kolmogorov-Smirnov method. Intergroup comparison was assessed by repeated measures ANOVA with Bonferroni correction or Kruskal-Wallis test. Intra-group differences were compared with paired Student's t-tests or Mann-Whitney test. Data was analyzed with SPSS software (version 22.0; IBM Corp., Armonk, NY, USA), and plotted with GraphPad Prism (version 7.0; GraphPad Software Inc., La Jolla, CA, USA). A value of $p < 0.05$ was considered statistically significant. Hypothesis tests were 2-sided.

© 2020 He X et al. *JAMA Network Open.*

eFigure 1. Characterization of hUC-MSCs

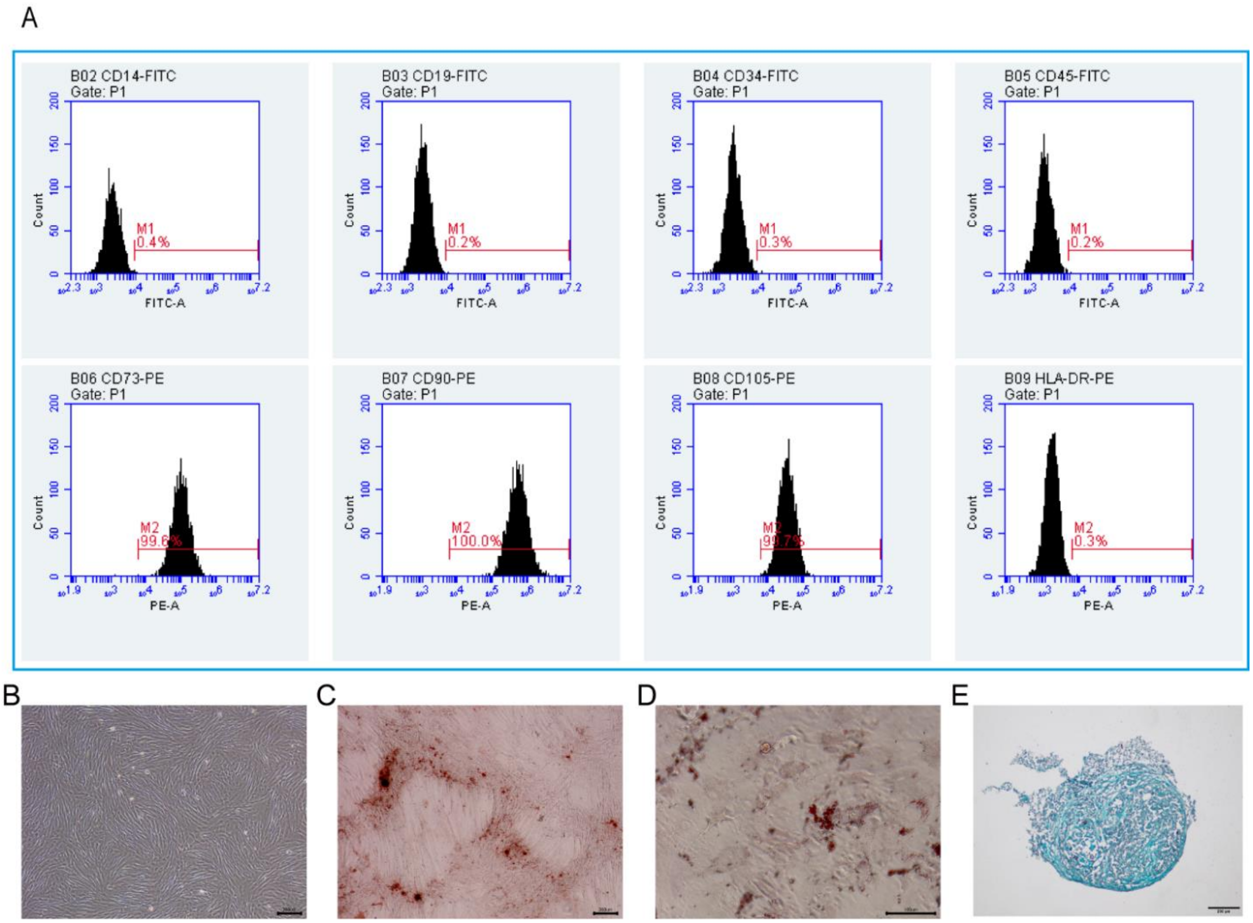

A, High expression of typical MSC markers CD73, CD90, and CD105 with a lack of the expression of CD14, CD19, CD34, CD45, and HLA-DR, as assayed by flow cytometry. B-E, Cells imaged with an ordinary optical microscope and the potential for bone, adipocyte and cartilage differentiation.

© 2020 He X et al. *JAMA Network Open*.

eFigure 2. Laboratory Results of Safety Assessment

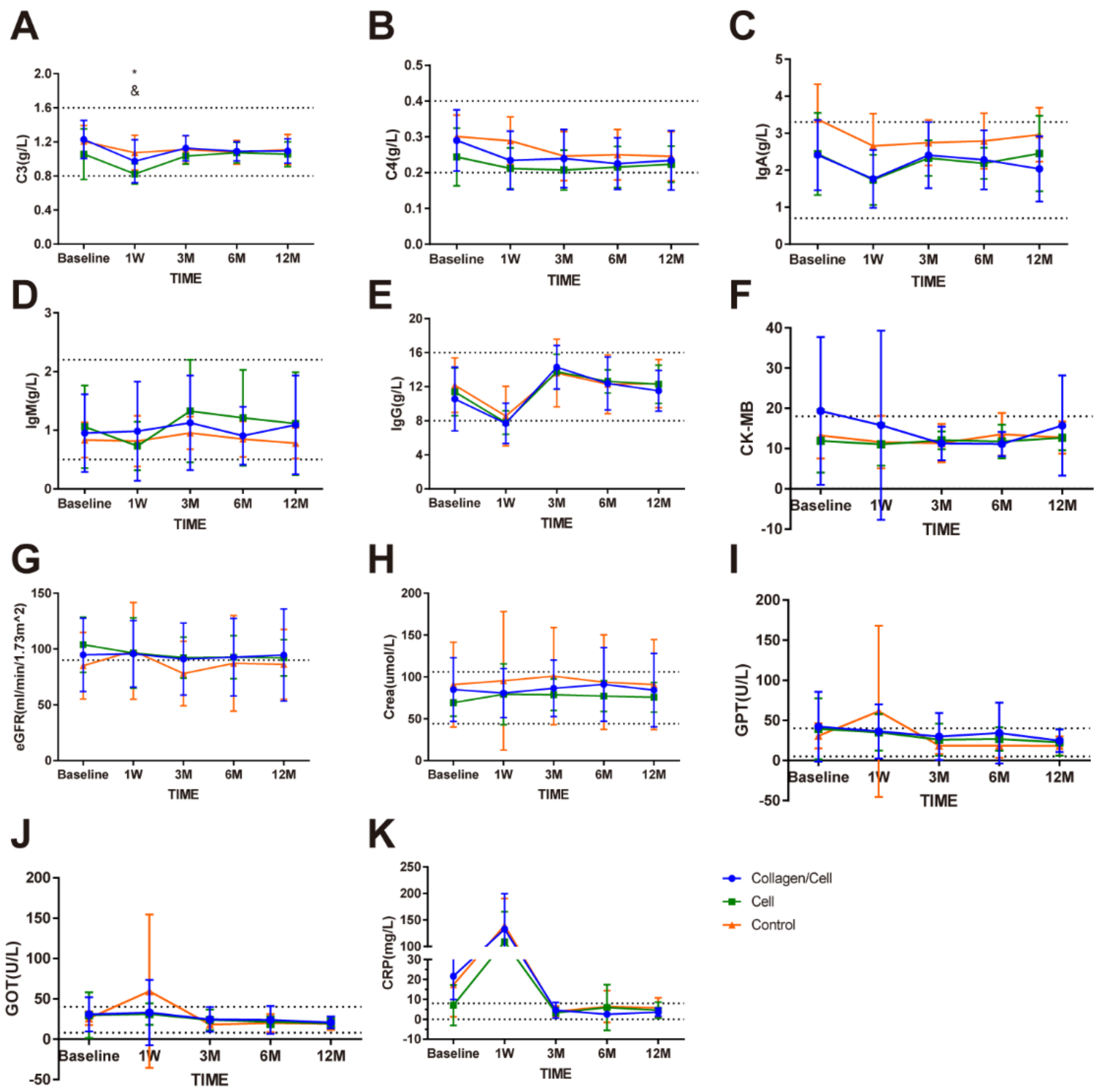

The laboratory results indicated that there were no significant differences in immunoglobulin (B-E), myocardial damage markers CK-MB (F), renal function (G & H), liver function (I & J), and CRP (K) among all groups pre- and post-operation. Broken lines gave normal range for all markers: C3, 0.8 - 1.6 g/L; C4, 0.2 - 0.4 g/L; IgA, 0.7 - 3.3 g/L; IgM, 0.5 - 2.2 g/L; IgG, 8 - 16 g/L; CK-MB, 0 - 18 U/L; eGFR, > 90 ml/min/1.73m^2; Crea, 44 - 106 umol/L; GPT, 5 - 40 U/L; GOT, 8 - 40 U/L; CRP, 0 - 8 mg/L. C3: alexin C3; C4: alexin C4; Crea: creatinine; eGFR: glomerular filtration rate; GPT: glutamic-pyruvic transaminase; GOT: glutamic oxalacetic transaminase; CRP: C-reactive protein. Graphs represent mean values. *P ≤ 0.05, cell group vs control group: & p ≤ 0.05. ANOVA or Kruskal-Wallis, post hoc multiple comparisons with Bonferroni correction.

© 2020 He X et al. *JAMA Network Open.*

eFigure 3. Restoration of Cardiac Function and Attenuation of Negative LV Remodeling

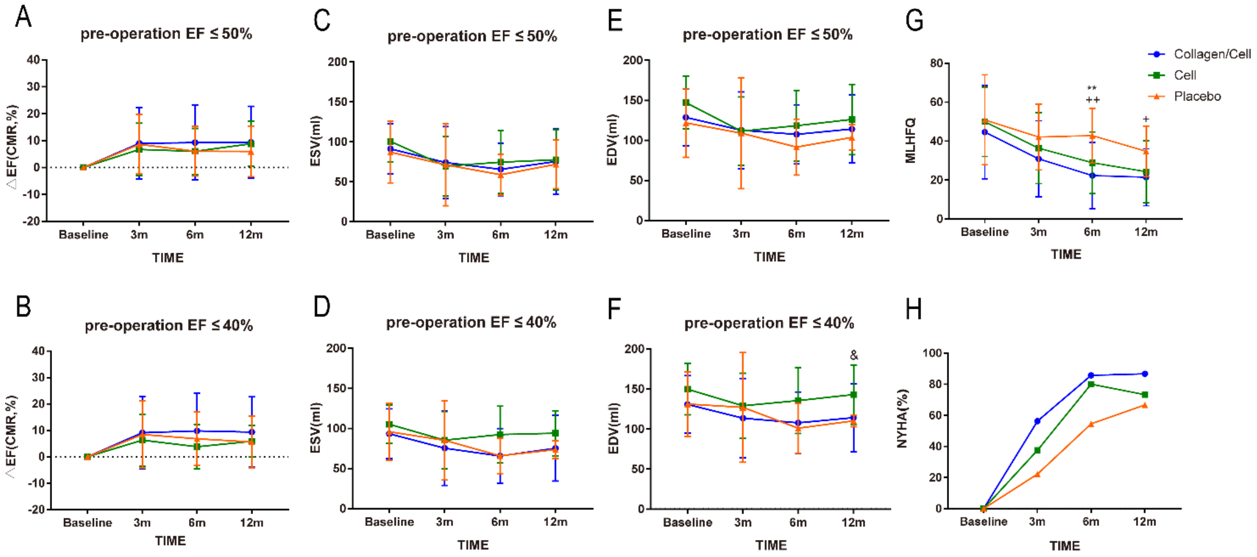

The cardiac magnetic resonance data suggested that cardiac function was recovered at 3 months, 6 months and 12 months after treatment. The collagen/cell group had a higher mean value for the LVEF than the other groups (A), which was especially obvious for patients with a pre-operative EF ≤ 40% (B). LVESV and LVEDV were reduced in all groups of patients, indicating a trend of negative left ventricular remodeling attenuation (C-F). The MLHFQ score improved compared with baseline in the collagen/cell group and cell group at 6 months and 12 months after treatment. In the control group, significant improvement was only observed 12 months later (G). There were more patients in the collagen/cell group with an improved NYHA class compared with the cell group and control group (H), y axis means percentage of patients with improved NYHA class in total patients.

Graphs represent the mean ± standard deviation. & p (cell group vs control group) ≤ 0.05, *P ≤ 0.05, **P ≤ 0.001, ANOVA or Kruskal-Wallis; ++ p (collagen/cell group vs control group) ≤ 0.01, post hoc multiple comparisons with Bonferroni correction.

© 2020 He X et al. *JAMA Network Open.*

eFigure 4. Blood Cell Count and Body Weight Change

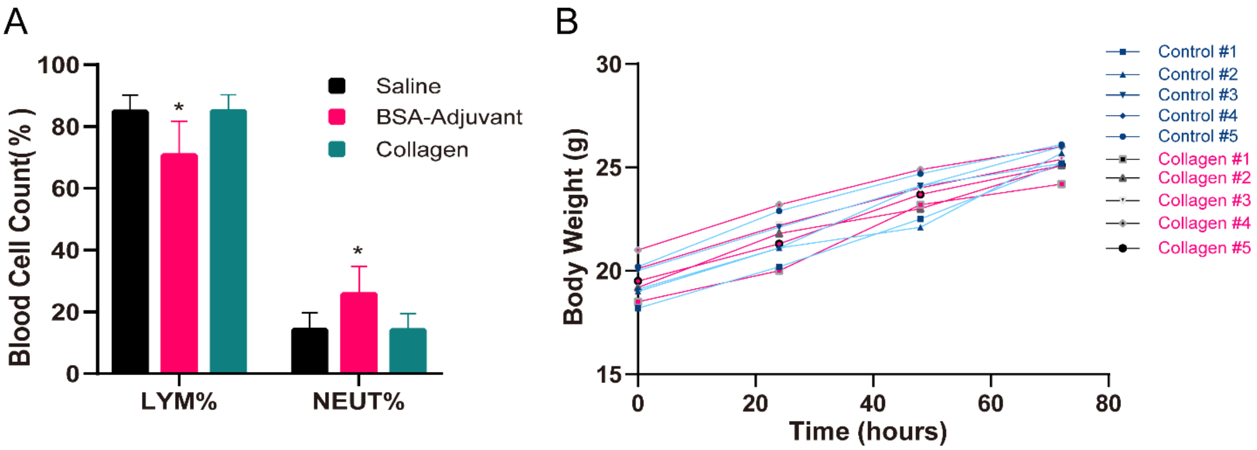

A, Blood cell count of mice after collagen hydrogel injection. BSA-adjuvant was used as positive control. Saline was used as negative control. The change of IgG and IgM were similar between saline and collagen hydrogel. B, The body weight change of mice in acute toxicity assay during 72 hours.

© 2020 He X et al. *JAMA Network Open.*

eFigure 5. Representative Cardiac Magnetic Resonance Images

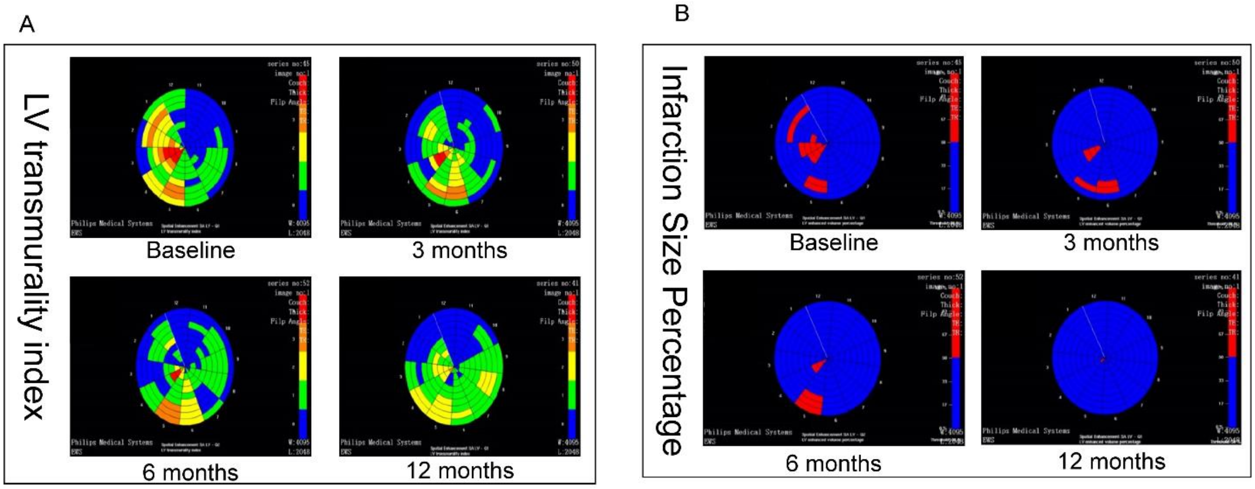

Representative cardiac magnetic resonance images showed a representative patient with significant reduction in myocardial transmurality (A) and nearly all transmural scar segments changed to non-transmural in the collagen/cell group (B). In panel A, segment colors from blue to red means transmurality from small to large Scar size. In panel B, segments with red color means more than half of the segments was infarct tissue.

© 2020 He X et al. *JAMA Network Open.*

eFigure 6. Analysis of Immune Cells in Mouse Spleen After Collagen Hydrogel Analysis

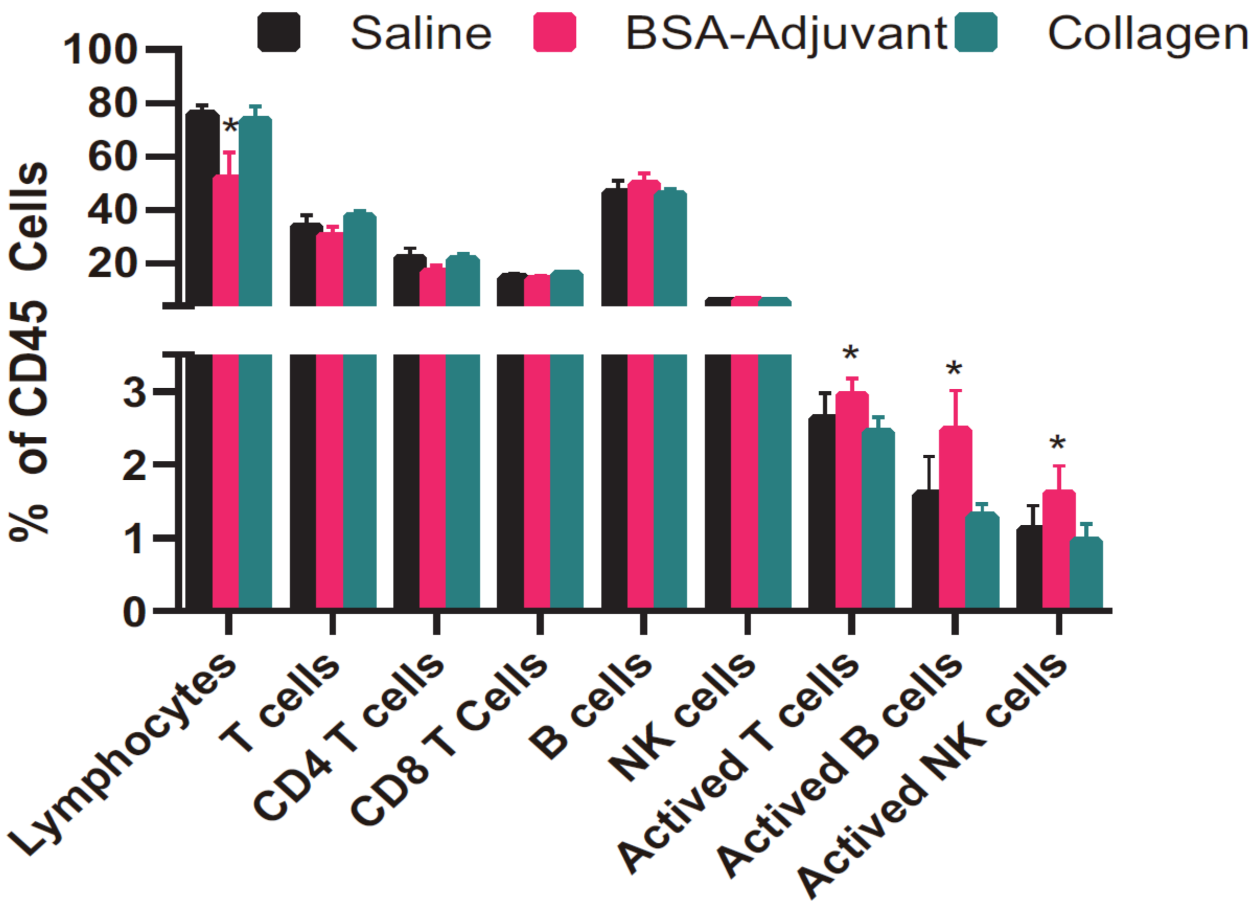

The percentage of different cells among CD45 positive cells was listed. T cells were identified by CD3 antibodies. B cells were identified by CD19 antibodies. NK cells were identified by CD49b. Activated T cells were identified by CD3 and CD69 antibodies. Activated B cells were identified by CD19 and CD69 antibodies. Activated NK cells were identified by CD49b and CD69 cells. BSA-adjuvant was used as positive control. Saline was used as negative control. The change of immune cells was similar between saline and collagen hydrogel.

© 2020 He X et al. *JAMA Network Open.*

eTable 1. CMR and MLHFQ Data (Safety Analysis Set)

	collagen/cell group	cell group	control group	p
Scar Size Percentage				
Baseline	13.18% ± 7.66%	15.17% ± 9.91%	14.00% ± 6.64%	0.813
3 months	13.33% ± 9.85%	11.18%* ± 8.79%	13.13% ± 8.64%	0.719
6 months	10.59%* ± 5.97%	13.62% ± 8.18%	16.83% ± 14.13%	0.594
12 months	9.41%* ± 7.2%	21.11% ± 12.43%	22.41% ± 15.11%	0.014
Scar Size (cm3)				
Baseline	7.40 ± 4.56	8.64 ± 4.47	8.77 ± 4.20	0.684
3 months	8.40 ± 7.03	7.05* ± 5.77	7.15 ± 5.94	0.918
6 months	6.56* ± 4.08	9.31 ± 5.10	17.53 ± 19.68	0.355
12 months	8.68 ± 13.40	20.08* ± 16.03	19.84* ± 12.13	0.017
Myocardium Size(cm3)				
Baseline	55.06 ± 11.75	60.24 ± 14.99	63.80 ± 23.06	0.416
3 months	58.34 ± 13.62	61.03 ± 16.26	49.03 ± 21.16	0.618
6 months	58.95 ± 9.53	65.31 ± 16.71	69.61 ± 34.48	0.499
12 months	67.55 ± 38.64	84.85 ± 37.06	92.88* ± 45.57	0.156
Viable Size(cm3)				
Baseline	47.66 ± 10.63	51.60 ± 16.05	54.75 ± 21.19	0.54
3 months	49.94 ± 11.20	53.98 ± 17.31	41.39 ± 18.73	0.562
6 months	52.38 ± 7.63	55.99 ± 16.31	53.02 ± 20.70	0.831
12 months	59.07* ± 26.12	64.78 ± 25.17	73.04 ± 40.79	0.549
EDV(ml)				
Baseline	128.80 ± 35.6	147.29 ± 32.8	121.84 ± 42.95	0.182
3 months	112.73 ± 47.87	111.59* ± 42.7	109.02 ± 69.21	0.986
6 months	107.61* ± 37.03	118.41* ± 44.04	91.68* ± 34.88	0.268
12 months	114.29* ± 42.5	126.17 ± 43.91	103.77* ± 15.83	0.336
ESV(ml)				
Baseline	91.16 ± 31.32	100.34 ± 25.33	86.90 ± 38.54	0.526
3 months	73.91 ± 45.06	69.39* ± 37.48	71.08 ± 51.29	0.967
6 months	65.35** ± 32.9	74.43* ± 39.24	58.74* ± 25.41	0.528
12 months	75.33* ± 40.87	77.47* ± 37.51	66.95* ± 18.18	0.728
CMR LVEF				
Baseline	30.13% ± 7.57%	33.05% ± 8.19%	30.61% ± 9.09%	0.572
3 months	39.87%* ± 16.54%	41.38%± 12.93%	38.24%* ± 12.34%	0.907
6 months	39.86%* ± 14.58%	39.85%* ± 13.01%	37.03% ± 6.86%	0.815
12 months	38.73%* ± 15.05%	41.44%** ± 13.75%	36.60% ± 10.05%	0.657
CMR LVEF(pre-operation EF < 40%)				
Baseline	29.37% ± 7.18%	29.63% ± 6.03%	27.98% ± 7.39%	0.836
3 months	39.31%* ± 17.01%	36. 65% ± 11.76%	34.10% ± 10.62%	0.5
6 months	39.59%* ± 15.14%	32.96% ± 8.96%	34.92% ± 5.61%	0.759
12 months	38.73%* ± 15.05%	34.62%** ± 6.87%	33.30% ± 6.9%	0.464
MLHFQ				

© 2020 He X et al. *JAMA Network Open.*

Baseline	44.63 ± 24.01	49.94 ± 17.82	50.92 ± 23.11	0.698
3 months	30.93 ± 19.53	36.38** ± 18.22	42.09 ± 16.92	0.321
6 months	22.29** ± 16.97	28.87** ± 15.81	42.82 ± 14.1	0.009
12 months	21.40* ± 14.65	24.27** ± 16	34.75** ± 12.98	0.064

Data are the number (%) or mean ± standard deviation. P, compared among groups, ANOVA or Kruskal-Wallis; * $p \leqslant 0.05$ (compared with baseline in each group), ** $p \leqslant 0.01$ (compared with baseline in each group), paired-sample T test or Mann-Whitney test.

CMR: cardiac magnetic resonance; LVEF: left ventricular ejection fraction; EDV: end-diastolic volume; ESV: end-systolic volume; MLHFQ: Minnesota Living with Heart Failure Questionnaire

eTable 2. Parameters of CABG Operation

	Collagen/hUC-MSCs (n=16)	hUC-MSCs (n=16)	Control (n=12)	p
**Operation time (minutes)**	366.56 ± 61.45	363.00 ± 46.24	405.42 ± 65.35	0.125
**LIMA**	16	16	12	1
**Total bypass count**				
**3**	3 (18.8%)	6 (37.5%)	3 (25%)	0.62
**4**	11 (68.8%)	7 (43.8%)	8 (66.7%)	
**5**	2 (12.5%)	3 (18.8%)	1 (8.3%)	
**Off-pump bypass**	15 (93.8%)	15 (93.8%)	10 (83.3%)	0.564
**ventricular aneurysm suture**	2 (12.5%)	3 (18.8%)	2 (16.7%)	0.887
**ventricular aneurysm resection**	0	1 (6.3%)	1 (8.3%)	0.531

Data are the number (%) or mean ± standard deviation. LIMA, Left internal mammary artery.

© 2020 He X et al. *JAMA Network Open.*

eTable 3. Intradermal Irritation Assay

Rabbit	Extraction Medium	24hours				48hours				72hours			
		Control		Collagen		Control		Collagen		Control		Collagen	
		ER	ED	ER	ED	ER	ED	ER	ED	ER	ED	ER	ED
**#1**	**Saline**	0	0	0	0	0	0	0	0	0	0	0	0
**#2**	**Saline**	0	0	0	0	0	0	0	0	0	0	0	0
**#3**	**Saline**	0	0	0	0	0	0	0	0	0	0	0	0
**#4**	**Oil**	2	2	1	2	2	2	1	2	2	1	1	1
**#5**	**Oil**	1	1	2	1	1	1	2	1	1	1	1	1
**#6**	**Oil**	2	2	2	2	2	1	2	1	1	1	1	1

ER=Erythema; ED=edema.

© 2020 He X et al. *JAMA Network Open.*

eTable 4. Inclusion Criteria

1	Male or female, 35-80 years old.
2	Coronary artery disease detected by coronary angiography not eligible for percutaneous coronary intervention.
3	MRI confirmation of coronary artery disease and ischemic regions.
4	LVEF ≤45% (assessed by 3D echocardiography).
5	NYHA Class II-IV.
6	No organ dysfunction in the lung, liver or kidney.
7	Patients are able and willing to observe the therapeutic effects and adverse events.
8	Signed informed consent was obtained.
9	Negative serum pregnancy test.
10	No coagulation dysfunction.
11	Glycated hemoglobin ≤ 6.5.

© 2020 He X et al. *JAMA Network Open*.

eTable 5. Exclusion Criteria

1	Patients with ACS, including non-ST-segment elevation myocardial infarction (NSTEMI) and ST-segment elevation myocardial infarction (STEMI).
2	Lactating or pregnant woman.
3	Ineligibility for CABG.
4	Unexplainable baseline laboratory abnormalities.
5	Sensitivity to any of the study medications.
6	Patients suffering from CVD, such as aortic disease, malignant arrhythmias, congenital heart disease, intra-cardiac mass, or moderate or severe valvular stenosis or regurgitation.
7	History of life threatening allergic or immune-mediated reaction.
8	Systemic infection or severe local infection.
9	Shock, MODS or an inability to cooperate with doctors.
10	Severe heart, lung, liver or renal dysfunction.
11	Use of a medicine that might have an effect on the assessed outcomes.
12	Suffering from HIV, Hepatitis B or Hepatitis C.
13	Participation in any clinical trial in the past three months.
14	History of mental illness or suicide risk.
15	High expectation or unrealistic demands.
16	Recent exposure to high levels of radiation.
17	A previous or current history of neoplasia or other comorbidities that could impact the patient's short-term survival.
18	Patients with serious complications of coronary artery disease (e.g., perforation of the interventricular septum, ventricular aneurysm or mitral regurgitation due to papillary muscle dysfunction).
19	Abnormal coagulation function.
20	Patients with hemodynamic instability that may lead to serious complications.
21	Any condition that, in the judgment of the investigator, would place the patient at risk.

© 2020 He X et al. *JAMA Network Open*.
