# Supplementary material for: Effect of Intramyocardial Grafting Collagen Scaffold With Mesenchymal Stromal Cells in Patients With Chronic Ischemic Heart Disease: A Randomized Clinical Trial
Source: JAMA Netw Open. 2020 Sep 10;3(9):e2016236. doi: 10.1001/jamanetworkopen.2020.16236 (PMC7489863; doi:10.1001/jamanetworkopen.2020.16236)
Supplement: Supplement 3. — Data Sharing Statement [file jamanetwopen-e2016236-s003.pdf]

Data Sharing Statement

He. Effect of Intramyocardial Grafting Collagen Scaffold With Mesenchymal Stromal Cells in Patients With Chronic Ischemic Heart Disease. *JAMA Netw Open*. Published September 10, 2020. 10.1001/jamanetworkopen.2020.16236

Data
Data available: No

Additional Information
Explanation for why data not available: The data used to support the findings of this study are available from the corresponding author upon request.
